# Zinc Oxide—From Synthesis to Application: A Review

**DOI:** 10.3390/ma7042833

**Published:** 2014-04-09

**Authors:** Agnieszka Kołodziejczak-Radzimska, Teofil Jesionowski

**Affiliations:** Institute of Chemical Technology and Engineering, Faculty of Chemical Technology, Poznan University of Technology, M. Sklodowskiej-Curie 2, PL-60965 Poznan, Poland; E-Mail: teofil.jesionowski@put.poznan.pl

**Keywords:** zinc oxide, synthesis, modification, application

## Abstract

Zinc oxide can be called a multifunctional material thanks to its unique physical and chemical properties. The first part of this paper presents the most important methods of preparation of ZnO divided into metallurgical and chemical methods. The mechanochemical process, controlled precipitation, sol-gel method, solvothermal and hydrothermal method, method using emulsion and microemulsion enviroment and other methods of obtaining zinc oxide were classified as chemical methods. In the next part of this review, the modification methods of ZnO were characterized. The modification with organic (carboxylic acid, silanes) and inroganic (metal oxides) compounds, and polymer matrices were mainly described. Finally, we present possible applications in various branches of industry: rubber, pharmaceutical, cosmetics, textile, electronic and electrotechnology, photocatalysis were introduced. This review provides useful information for specialist dealings with zinc oxide.

## 1. Introduction

Zinc oxide, with its unique physical and chemical properties, such as high chemical stability, high electrochemical coupling coefficient, broad range of radiation absorption and high photostability, is a multifunctional material [1,2]. In materials science, zinc oxide is classified as a semiconductor in group II-VI, whose covalence is on the boundary between ionic and covalent semiconductors. A broad energy band (3.37 eV), high bond energy (60 meV) and high thermal and mechanical stability at room temperature make it attractive for potential use in electronics, optoelectronics and laser technology [3,4]. The piezo- and pyroelectric properties of ZnO mean that it can be used as a sensor, converter, energy generator and photocatalyst in hydrogen production [5,6]. Because of its hardness, rigidity and piezoelectric constant it is an important material in the ceramics industry, while its low toxicity, biocompatibility and biodegradability make it a material of interest for biomedicine and in pro-ecological systems [7–9].

**Figure 1.**
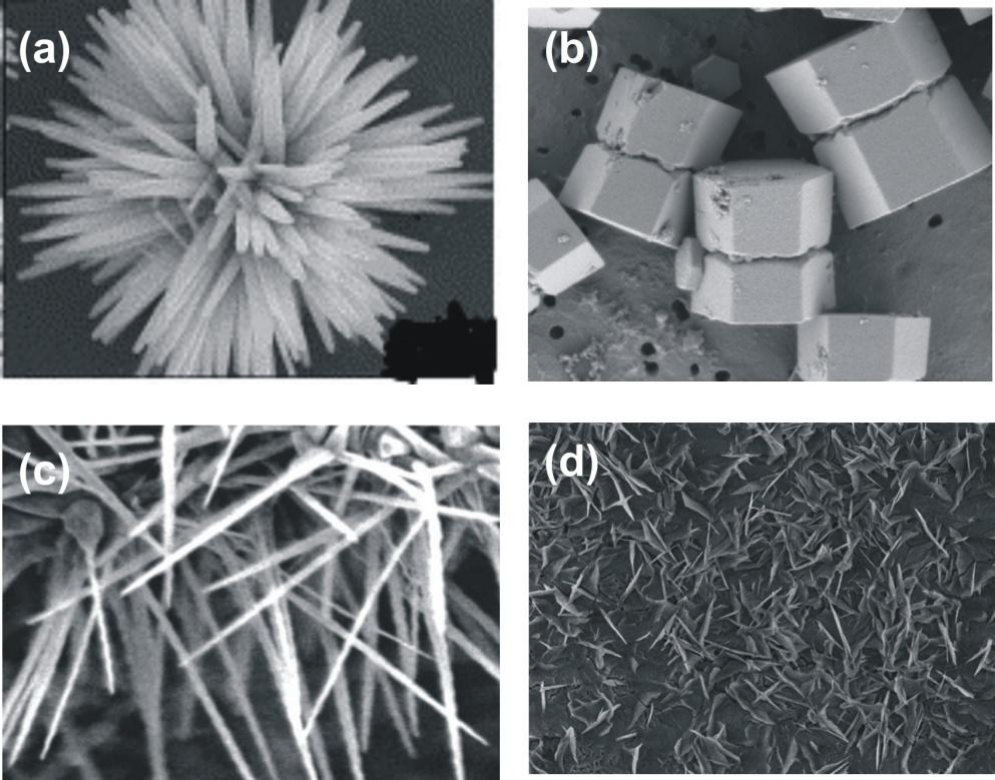
Examples of zinc oxide structure: flower (**a**); rods (**b**); wires (**c**,**d**) (created based on [17,27,29] with permission from Elsevier Publisher and [11] AIP Publishing LLC).

The variety of structures of nanometric zinc oxide means that ZnO can be classified among new materials with potential applications in many fields of nanotechnology. Zinc oxide can occur in one- (1D), two- (2D), and three-dimensional (3D) structures. One-dimensional structures make up the largest group, including nanorods [10–12], -needles [13], -helixes, -springs and -rings [14], -ribbons [15], -tubes [16–18] -belts [19], -wires [20–22] and -combs [23]. Zinc oxide can be obtained in 2D structures, such as nanoplate/nanosheet and nanopellets [24,25]. Examples of 3D structures of zinc oxide include flower, dandelion, snowflakes, coniferous urchin-like, *etc.* [26–29]. ZnO provides one of the greatest assortments of varied particle structures among all known materials (see Figure 1).

In this review, the methods of synthesis, modification and application of zinc oxide will be discussed. The zinc oxide occurs in a very rich variety of structures and offers a wide range of properties. The variety of methods for ZnO production, such as vapour deposition, precipitation in water solution, hydrothermal synthesis, the sol-gel process, precipitation from microemulsions and mechanochemical processes, makes it possible to obtain products with particles differing in shape, size and spatial structure. These methods are described in detail in the following sections (Table 1).

## 2. Methods of Synthesis of Nano- and Micrometric Zinc Oxide

### 2.1. Metallurgical Process

Metallurgical processes for obtaining zinc oxide are based on the roasting of zinc ore. According to the ISO 9298 standard [68], zinc oxide is classified either as type A, obtained by a direct process (the American process); or type B, obtained by an indirect process (the French process).

The direct (American) process involves the reduction of zinc ore by heating with coal (such as anthracite), followed by the oxidation of zinc vapour in the same reactor, in a single production cycle. This process was developed by Samuel Wetherill, and takes place in a furnace in which the first layer consists of a coal bed, lit by the heat remaining from the previous charge. Above this bed is a second layer in the form of zinc ore mixed with coal. Blast air is fed in from below, so as to deliver heat to both layers and to carry carbon monoxide for zinc reduction. The resulting zinc oxide (of type A) contains impurities in the form of compounds of other metals from the zinc ore. The resulting ZnO particles are mainly needle-shaped, and sometimes spheroidal. To obtain a product with a permanent white color, the oxides of lead, iron and cadmium that are present are converted to sulfates. Increasing the permanence of the color is linked to increasing the content of water-soluble substances, and also increasing the acidity of the product. Acidity is desirable in the case of rubber processing technology, since it lengthens prevulcanization time and ensures the safe processing of the mixtures [69].

In the indirect (French) process, metallic zinc is melted in a furnace and vaporized at *ca.* 910 °C. The immediate reaction of the zinc vapour with oxygen from the air produces ZnO. The particles of zinc oxide are transported via a cooling duct and are collected at a bag filter station. The indirect process was popularized by LeClaire in 1844, and since then has been known as the French process. The product consists of agglomerates with an average particle size ranging from 0.1 to a few micrometres [70]. The ZnO particles are mainly of spheroidal shape. The French process is carried out in vertical furnaces, with an original vertical charge, vertical refining column, vaporizer with electric arc, and rotary combustion chamber [71]. Type B zinc oxide has a higher degree of purity than type A.

### 2.2. Chemical Processes

Because of its interesting properties, zinc oxide has been the subject of study by many researchers. This has led to the development of a great variety of techniques for synthesizing the compound. Unfortunately, methods that work in the laboratory cannot always be applied on an industrial scale, where it is important for the process to be economically effective, high yielding and simple to implement.

#### 2.2.1. Mechanochemical Process

The mechanochemical process is a cheap and simple method of obtaining nanoparticles on a large scale. It involves high-energy dry milling, which initiates a reaction through ball–powder impacts in a ball mill, at low temperature. A “thinner” is added to the system in the form of a solid (usually NaCl), which acts as a reaction medium and separates the nanoparticles being formed. A fundamental difficulty in this method is the uniform grinding of the powder and reduction of grains to the required size, which decreases with increasing time and energy of milling. Unfortunately, a longer milling time leads to a greater quantity of impurities. The advantages of this method are the low production costs, small particle sizes and limited tendency for particles to agglomerate, as well as the high homogeneity of the crystalline structure and morphology.

The starting materials used in the mechanochemical method are mainly anhydrous ZnCl_2_ and Na_2_CO_3_. NaCl is added to the system; this serves as a reaction medium and separates the nanoparticles. The zinc oxide precursor formed, ZnCO_3_, is calcined at a temperature of 400–800 °C. The process as a whole involves the following reactions (1) and (2):

$${\text{ZnCl}}_{2}+{\text{Na}}_{2}{\text{CO}}_{3}\to {\text{ZnCO}}_{3}+2\text{NaCl}$$ (1)

$${\text{ZnCO}}_{\text{3}}\overset{\text{temperature}}{\to }\text{ZnO}+{\text{CO}}_{\text{2}}$$ (2)

The mechanochemical method was proposed by Ao *et al.* [30], they synthesized ZnO with an average crystallite size of 21 nm. The milling process was carried out for 6 h, producing ZnCO_3_ as the zinc oxide precursor. Calcination of the precursor at 600 °C produced ZnO with a hexagonal structure. Tests showed that the size of the ZnO crystallites depends on the milling time and calcination temperature. Increasing the milling time (2–6 h) led to a reduction in the crystallite sizes (21.5–25 nm), which may indicate the existence of a “critical moment”. Meanwhile an increase in the calcination temperature from 400 to 800 °C caused an increase in crystallite size (18–35 nm).

The same system of reagents was used by Tsuzuki and McCormick [32]. They found that a milling time of 4 h was enough for a reaction to take place between the substrates, producing the precursor ZnCO_3_, which when calcined at 400 °C produced nanocrystallites of ZnO with an average size of 26 nm. Tsuzuki *et al.* showed that milling of the substrates without a thinner leads to the formation of aggregates measuring 100–1000 nm. This confirmed the important role played by zinc chloride in preventing agglomeration of the nanoparticles.

A milling process of ZnCl_2_ and Na_2_CO_3_ was also carried out by Moballegh *et al.* [33] and by Aghababazadeh *et al.* [34]. Moballegh *et al.*, investigated the effect of calcination temperature on particle size. An increase in the temperature of the process (300–450 °C) caused an increase in the size of the ZnO particles (27–56 nm). Aghababazadeh *et al.* obtained ZnO with an average particle size of approximately 51 nm and a surface area of 23 m^2^/g, carrying out the process at a temperature of 400 °C.

Stanković *et al.* [31] extended their previous study to investigate mechanical-thermal synthesis (MTS)—mechanical activation followed by thermal activation of ZnO from ZnCl_2_ and oxalic acid (C_2_H_2_O_4_·2H_2_O) as reactants with the intention of obtaining pure ZnO nanopowder. The study also aimed to examine the effects of oxalic acid as an organic PCA, and different milling times, on the crystal structure, average particle size and morphology of ZnO nanopowders. The mixture of initial reactants was milled from 30 min up to 4 h, and subsequently annealed at 450 °C for 1 h. Qualitative analysis of the prepared powders was performed using X-ray diffraction (XRD) and Raman spectroscopy. The XRD analysis showed perfect long-range order and the pure wurtzite structure of the synthesized ZnO powders, irrespective of the milling duration. By contrast, Raman spectroscopy indicates a different middle-range order of ZnO powders. From the SEM images, it is observed that the morphology of the particles strongly depends on the milling time of the reactant mixture, regardless of the further thermal treatment. A longer time of milling led to a smaller particle size.

#### 2.2.2. Controlled Precipitation

Controlled precipitation is a widely used method of obtaining zinc oxide, since it makes it possible to obtain a product with repeatable properties. The method involves fast and spontaneous reduction of a solution of zinc salt using a reducing agent, to limit the growth of particles with specified dimensions, followed by precipitation of a precursor of ZnO from the solution. At the next stage this precursor undergoes thermal treatment, followed by milling to remove impurities. It is very difficult to break down the agglomerates that form, so the calcined powders have a high level of agglomeration of particles. The process of precipitation is controlled by parameters such as pH, temperature and time of precipitation.

Zinc oxide has also been precipitated from aqueous solutions of zinc chloride and zinc acetate [35]. Controlled parameters in this process included the concentration of the reagents, the rate of addition of substrates, and the reaction temperature. Zinc oxide was produced with a monomodal particle size distribution and high surface area.

A controlled precipitation method was also used by Hong *et al.* [36]. The process of precipitating zinc oxide was carried out using zinc acetate (Zn(CH_3_COO)_2_·H_2_O) and ammonium carbonate (NH_4_)_2_CO_3_. These solutions were dosed into a vigorously mixed aqueous solution of poly(ethylene glycol) with an average molecular mass of 10,000. The resulting precipitate was calcined by two different methods. In the first, calcination at 450 °C for 3 h produced ZnO labelled as “powder A”. In the second process, calcination took place following heterogeneous azeotropic distillation of the precursor; the resulting zinc oxide was labelled as “powder B”. Structural testing (XRD) and morphological analysis (TEM) showed that powder A contained particles with a diameter of 40 nm, while powder B contained particles with a diameter of 30 nm. Heterogeneous azeotropic distillation completely reduces the occurrence of agglomerates and decreases the ZnO particle size.

Lanje *et al.* [38] used the cost competitive and simple precipitation process for the synthesis of zinc oxide. The single step process with the large scale production without unwanted impurities is desirable for the cost-effective preparation of ZnO nanparticles. As a consequence, the low cost precursors such as zinc nitrate and sodium hydroxide to synthesize the ZnO nanoparticles (*ca.* 40 nm) were used. In order to reduce the agglomeration among the smaller particles, the starch molecule which contains many O-H functional groups and could bind surface of nanoparticles in initial nucleation stage, was used.

Another process of controlled precipitation of zinc oxide was carried out by Wang *et al.* [39]. Nanometric zinc oxide was obtained by precipitation from aqueous solutions of NH_4_HCO_3_ and ZnSO_4_·7H_2_O by way of the following reactions (3) and (4):

$$5{\text{ZnSO}}_{4(\text{aq})}+10{\text{NH}}_{4}{\text{HCO}}_{3(\text{aq})}\to {\text{Zn}}_{5}{\left({\text{CO}}_{3}\right)}_{2}{\left(\text{OH}\right)}_{6(s)}+5{\left({\text{NH}}_{4}\right)}_{2}{\text{SO}}_{4(\text{aq})}+8{\text{CO}}_{2(g)}+2H_{2}O_{\left(l\right)}$$ (3)

$${\text{Zn}}_{5}{\left({\text{CO}}_{\text{3}}\right)}_{2}{\left(\text{OH}\right)}_{6(s)}\to {\text{5ZnO}}_{\left(s\right)}+2{\text{CO}}_{2(g)}+3H_{2}O_{\left(g\right)}$$ (4)

This study was performed using a membrane reactor consisting of two plates of polytetrafluoroethylene (PTFE), with stainless steel as a dispersion medium. The ZnO obtained had a narrow range of particle sizes, from 9 to 20 nm. XRD analysis showed both the precursor and the ZnO itself to have a wurtzite structure exclusively. The particle size was affected by temperature, calcination time, flow rate and concentration of the supply phase.

In a report of Jia *et al.* [40], *in situ* crystallization transformation from Zn(OH)_2_ to ZnO is demonstrated. Based on observations using X-ray diffraction (XRD) and scanning electron microscopy (SEM), two possible mechanisms from Zn(OH)_2_ to ZnO are suggested. The formation mechanism of ZnO was studied in a time-resolved investigation by heating a water solution containing zinc salts (Zn(CH_3_COO)_2_) and ammonium hydroxide (NH_4_OH) to 85 °C. Transformation of microcrystals of the stable intermediate ε-Z(OH)_2_ to ZnO was observed to occur at various aging times. Transformation from ε-Z(OH)_2_ to ZnO followed two mechanisms: dissolution−reprecipitation and *in situ* crystallization transformation involving dehydration and internal atomic rearrangements. From a fundamental point of view, these findings provide new insights into the growth of ZnO crystals and arm researchers with potential strategies for the controllable synthesis of ZnO in liquid media.

In processes of synthesis of nanopowders based on precipitation, it is increasingly common for surfactants to be used to control the growth of particles. The presence of these compounds affects not only nucleation and particle growth, but also coagulation and flocculation of the particles. The surfactant method involves chelation of the metal cations of the precursor by surfactants in an aqueous environment. Wang *et al.* [44] obtained nanometric zinc oxide from ZnCl_2_ and NH_4_OH in the presence of the cationic surfactant CTAB (cetyltrimethylammonium bromide). The process was carried out at room temperature, and the resulting powder was calcined at 500 °C to remove residues of the surfactant. The product was highly crystalline ZnO with a wurtzite structure and with small, well-dispersed spherical nanoparticles in size of 50 nm. It was found that CTAB affects the process of nucleation and growth of crystallites during synthesis, and also prevents the formation of agglomerates.

**Figure 2.**
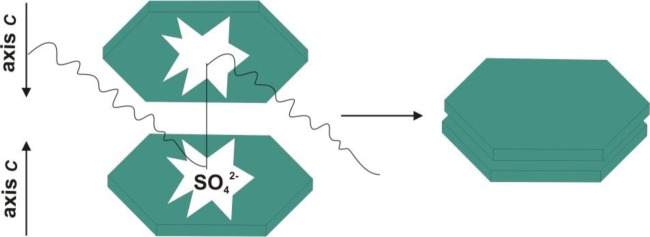
Effect of sodium dodecyl sulfate (SDS) surfactant on the structure of a ZnO crystal (created based on [45] with permission from Elsevier Publisher).

Li *et al.* [45] synthesized microcrystals of zinc oxide with various shapes (including forms resembling rice grains, nuts and rods) from Zn(NO_3_)_2_·6H_2_O and NaOH in the presence of sodium dodecyl sulfate (SDS) and triethanolamine (TEA) as cationic surfactant. The presence of the surfactant was found to affect both the shape and size of the resulting ZnO particles. Li *et al.* suggested additionally that the transformation may take place via a mechanism of recrystallization. Figure 2 shows the effect of SDS on the structure of the ZnO crystal.

#### 2.2.3. Sol-Gel Method

**Figure 3.**
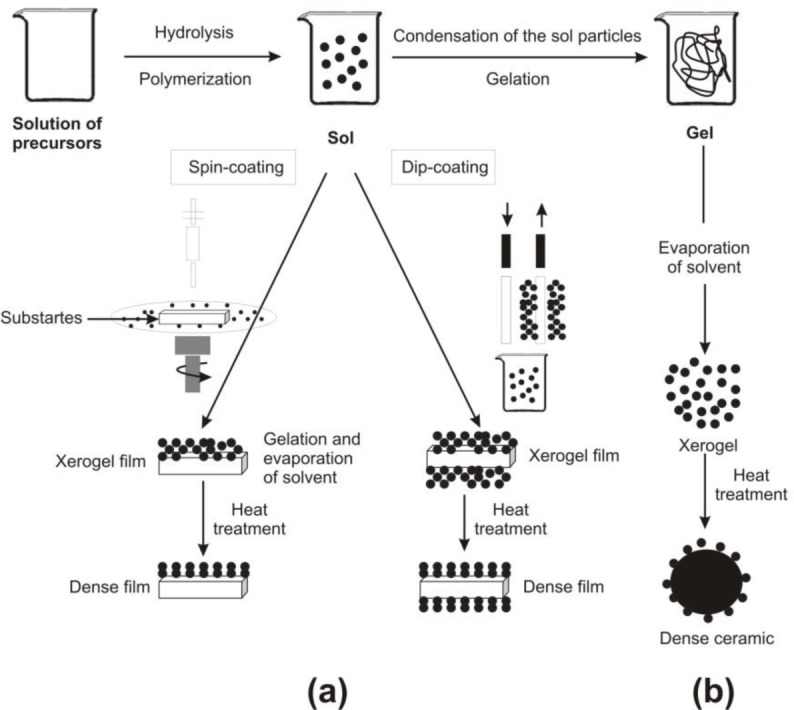
Overview showing two examples of synthesis by the sol-gel method: (**a**) films from a colloidal sol; (**b**) powder from a colloidal sol transformed into a gel (created based on [72] with permission from Elsevier Publisher).

The obtaining of ZnO nanopowders by the sol-gel method is the subject of much interest, in view of the simplicity, low cost, reliability, repeatability and relatively mild conditions of synthesis, which are such as to enable the surface modification of zinc oxide with selected organic compounds. This changes in properties and extends its range of applications. The favourable optical properties of nanoparticles obtained by the sol-gel method have become a common topic of research, as reflected in numerous scientific publications [46]. Figure 3 shows two examples of synthesis by the sol-gel method: films from a colloidal sol (Figure 3a), and powder from a colloidal sol transformed into a gel (Figure 3b).

Benhebal *et al.* [47] prepared ZnO powder by sol-gel method from zinc acetate dihydrate, oxalic acid, using ethanol as solvent. The obtained product was characterized by using techniques such as nitrogen adsorption isotherms, X-ray difration (XRD), scanning electron microscopy (SEM), an UV-Vis spectroscopy. The prepared zinc oxide has a hexagonal wurtzite structure with the particles of a spherically shaped. A surface area obtained by the *BET* method of the calcined ZnO powder is equal to 10 m^2^/g, characteristic of a material with low prosity, or a crystallized material.

The sol-gel method was also used to obtain nanocrystalline zinc oxide by Ristić *et al.* [48]. A solution of tetramethylammonium hydroxide (TMAH) was added to a solution of zinc 2-ethylhexanoate (ZEH) in propan-2-ol. The resulting colloidal suspension was left for 30 min (alternatively for 24 h), and was then washed with ethanol and water. TMAH is a strong organic base, which comparably with an inorganic base (e.g., NaOH) is characterized by a pH of ~14. This high pH means that metal oxides are not contaminated with the cation from the base, which may have an effect on the ohmic conductance of the oxide material. A determination was made of the effect of the quantity of ZEH used and the maturing time of the colloidal solution. TEM images showed that the ZnO particles obtained have sizes of the order of 20–50 nm. The quantity of ZEH has a negligible effect on the particle size.

Yue *et al.* [49] also obtained ZnO by the sol-gel method. High-filling, unifrom, ordered ZnO nanotubes have been successully prepared by sol-gel method into ultrathin AAO membrane. Integrating the ultrathin AAO membranes with the sol-gel technique may help to fabricate high-quality 1D nanomaterials and to extend its application as a template for nanostructures growth.

#### 2.2.4. Solvothermal and Hydrothermal Method

The hydrothermal method does not require the use of organic solvents or additional processing of the product (grinding and calcination), which makes it a simple and environmentally friendly technique. The synthesis takes place in an autoclave, where the mixture of substrates is heated gradually to a temperature of 100–300 °C and left for several days. As a result of heating followed by cooling, crystal nuclei are formed, which then grow. This process has many advantages, including the possibility of carrying out the synthesis at low temperatures, the diverse shapes and dimensions of the resulting crystals depending on the composition of the starting mixture and the process temperature and pressure, the high degree of crystallinity of the product, and the high purity of the material obtained [73,74].

An example of a hydrothermal reaction is the synthesis of zinc oxide as proposed by Chen *et al.* [50], using the reagents ZnCl_2_ and NaOH in a ratio of 1:2, in an aqueous environment. The process took place by way of reaction (5):

$${\text{ZnCl}}_{2}+2\text{NaOH}\to \text{Zn}{\left(\text{OH}\right)}_{2}↓+2{\text{Na}}^{+}+2{\text{Cl}}^{-}$$ (5)

The white Zn(OH)_2_ precipitate underwent filtration and washing, and then the pH was corrected to a value of 5–8 using HCl. In the autoclave hydrothermal heating takes place at a programmed temperature for a set time, followed by cooling. The end product of the process is zinc oxide the following reaction (6):

$$\text{Zn}{\left(\text{OH}\right)}_{2}\to \text{ZnO}+H_{2}O$$ (6)

The average size and the morphology of the resulting ZnO particles were analyzed using an X-ray diffractometer (XRD) and transmission electron microscope (TEM). The temperature and time of reaction were shown to have a significant effect on the structure and size of the ZnO particles. It was also found that as the pH of the solution increases, there is an increase in the crystallinity and size of the particles, which reduces the efficiency of the process.

A hydrothermal process was also used by Ismail *et al.* [51], who obtained zinc oxide by way of the following reactions (7) and (8):

$$\text{Zn}{\left({\text{CH}}_{3}\text{COO}\right)}_{2}+2\text{NaOH}\to \text{Zn}{\left(\text{OH}\right)}_{2}+2{\text{CH}}_{3}\text{COONa}$$ (7)

$$\text{Zn}{\left(\text{OH}\right)}_{2}\overset{\text{temperature}}{\to }\text{ZnO}+H_{2}O$$ (8)

The chemical reaction between Zn(CH_3_COO)_2_ and NaOH was carried out in the presence of hexamethylenetetramine (HMTA), at room temperature. The resulting precipitate of Zn(OH)_2_ was washed with water several times, and then underwent thermal treatment in a Teflon-lined autoclave. Based on SEM images, the authors concluded that the HTMA, as a surfactant, plays an important role in the modification of the ZnO particles. The shape of the particles is also affected by the time and temperature of the hydrothermal process. With an increase in time, temperature and surfactant concentration, the size of the particles increases. Hydrothermal processing of the precursor, followed by drying, produced spherical particles of ZnO with sizes in the range 55–110 nm depending on the conditions of synthesis.

Dem’Yanets *et al.* [52] used a hydrothermal method to synthesize nanocrystalline zinc oxide with different particle shapes and sizes. A reaction of zinc acetate or nitrate with a suitable hydroxide (LiOH, KOH, NH_4_OH) produced the precursor Zn(OH)_2_·*n*H_2_O. The process was carried out in an autoclave, in isothermal conditions or at variable temperature (120–250 °C). Dehydration of the precursor, followed by recrystallization, produced crystallites of ZnO with a hexagonal structure and sizes of 100 nm–20 μm. Increasing the time of the hydrothermal process caused an increase in the diameter of the ZnO particles. It was observed that an increase in temperature by 50–70 °C enabled a fourfold reduction in the time of the experiment, which is a very favourable phenomenon.

Musić *et al.* [53] determined the effect of chemical synthesis on the size and properties of ZnO particles. A suspension obtained from a solution of Zn(CH_3_COO)_2_·2H_2_O and neutralized using different quantities of a solution of NH_4_OH underwent hydrothermal treatment in an autoclave at a temperature of 160 °C. It was found that the pH affected the size and shape of the ZnO particles. Maturing of the original aqueous suspension for 7 months (at a pH of 10, and at room temperature) led to the appearance of aggregates consisting of ZnO particles with sizes between ~20 and ~60 nm. Musić *et al.*, also synthesized zinc oxideu sing a sol-gel method, involving rapid hydrolysis of zinc 2-ethylhexanoate dissolved in propan-2-ol. The resulting nanoparticles cause distinct changes in the standard Raman spectrum of zinc oxide.

A number of studies [54,55,75,76] have shown that the use of microwave reactors in hydrothermal synthesis processes brings significant benefits. Microwaves make it possible to heat the solutions from which the synthesis products are obtained, while avoiding loss of energy on heating the entire vessel. Many chemical syntheses proceed with greater speed and yield when microwaves are used than in the case of traditional methods. Similar fast and voluminal heating of the reaction substrates can be achieved using electrical current flowing through the substrates. Strachowski *et al.* [77,78] carried out a systematic study comparing the ZnO obtained in reactors with different methods of reaction stimulation. The work was conducted in such a way that the reactions being compared took place at the same externally supplied power levels and with the same reaction vessel geometry. Reactors were used with reaction energy supplied using microwaves, electrical current, Joule heating, high-voltage pulses, and heating of the whole autoclave. Strachowski *et al.* found that nanopowders with phase composition closest to pure ZnO were obtained through microwave synthesis and in the traditional autoclave. The powders produced in the other reactors showed the presence of other phases (simonkolleite and hydroxyzincite) besides zinc oxide. The use of a microwave reactor made it possible to shorten the reaction time several fold, and also produced the purest product.

Microwaves were also used by Schneider *et al.* [56]. Zinc oxide was obtained by heating, using microwaves, zinc acetylacetonate and a zinc oxime complex in various alkoxyethanols (methoxy-, ethoxy- and butoxyethanol). Schneider *et al.*, showed that the morphology and aggregation of ZnO particles depends strongly on the precursor used. The zinc oxide obtained was analyzed using such methods as DLS (dynamic light scattering), *BET* surface area, SEM, TEM, XRD, TG, PL spectra and EPR (electronparamagnetic resonance). The size of the particles of the final product lay in the range 40–200 nm, depending on which precursor and alcohol were used. The smallest particles belonged to the zinc oxide obtained by heating a complex of zinc oxime in methoxyethanol. With an increase in the concentration and chain length of the alcohol, the particle size increased. The surface area of the ZnO lay in the range 10–70 m^2^/g. Thermal decomposition of both zinc acetylacetonate and zinc oxime enabled the obtaining of a product with the desired properties.

Zhang *et al.* [55] obtained ZnO particles in the shape of spheres and hollow spheres through a solvothermal reaction, in the presence of an ionic liquid (imidazolium tetrafluoroborate). The authors suggested that the solvothermal process may involve the following reactions (9) and (10):

$$2(1-x){\text{OH}}^{-}+2xF^{-}+{\text{Zn}}^{2+}\to \text{Zn}{\left(\text{OH}\right)}_{2-2x}F_{2x}$$ (9)

$$\text{Zn}{\left(\text{OH}\right)}_{2-2x}F_{2x}\to {\text{ZnO}}_{1-x-y}F_{2x}{\left(\text{OH}\right)}_{2y}+(1-x-y)H_{2}O$$ (10)

The hollow spheres which were obtained had diameters of 2–5 μm and contained channels approximately 10 nm in diameter. The thickness of the wall of such a sphere was approximately 1 μm. The system proposed by Zhang *et al.* may combine the properties of both a solvothermal hybrid and an ionothermal system. It can be expected that a solvothermal hybrid and an ionothermal system may be successfully used to synthesize new materials with interesting properties and morphologies.

A solvothermal method was also used by Chen *et al.* [54], who prepared nanocrystalline ZnO, free of hydroxyl groups. It was obtained from a reaction of zinc powder with trimethylamine *N*-oxide (Me_3_N→O) and 4-picoline *N*-oxide (4-pic→NO), carried out in an environment of organic solvents (toluene, ethylenediamine (EDA) and *N,N,N′,N′*-tetramethylenediamine (TMEDA)), in an autoclave at 180 °C. The process involved the following reactions (11) and (14):

$$\text{Zn}+2H_{2}O\to \text{Zn}{\left(\text{OH}\right)}_{2}+2H•$$ (11)

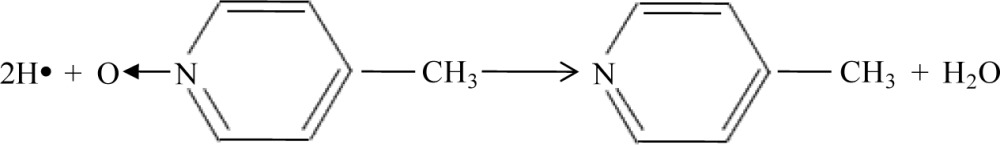
(12)

$${\text{Zn(OH)}}_{2}\to \text{ZnO}+H_{\text{2}}O$$ (13)

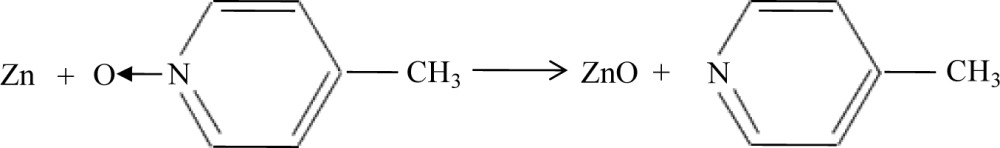
(14)

The oxidizing agents used and the coordinating abilities of the solvents affected the morphology and size of the nanoparticles/nanowires of ZnO. The authors also determined the effect of the presence of water in the reaction system. It was found that the presence of trace quantities of water catalyzed the reaction between zinc powder and 4-picN→O and affected the size of the ZnO nanocrystallites. The zinc oxide obtained had diameters in the range 24–185 nm, depending on the reaction conditions.

#### 2.2.5. Method Using an Emulsion or Microemulsion Environment

The classic definition of an emulsion as a continuous liquid phase in which is dispersed a second, discontinuous, immiscible liquid phase is far from complete. One very convenient way to classify emulsions is first to divide them into two large groups based on the nature of the external phase. The two groups are usually called *oil-in-water* (O/W) and *water-in-oil* (W/O) emulsions. The terms “oil” and “water” are very general; almost any highly polar, hydrophilic liquid falls into the “water” category in this definition, while hydrophobic, nonpolar liquids are considered “oils” [60,61].

Vorobyova *et al.* [58] used emulsion systems in their work. Zinc oxide was precipitated in an interphase reaction of zinc oleate (dissolved in decane) with sodium hydroxide (dissolved in ethanol or water). The process as a whole involved the reaction (15):

$$\text{Zn}{\left(C_{17}H_{33}\text{COO}\right)}_{2}(\text{decane})\text{+}2\text{NaOH}(\text{water and ethanol})\to \text{ZnO}+H_{2}O+2C_{17}H_{33}\text{COONa}$$ (15)

SEM and XRD analysis was performed on the ZnO powders obtained, following removal of the solvents and drying at room temperature. It was found that the reaction may take place in different phases, both in water and in the organic phase. The conditions of the process (temperature, substrates and ratio of two-phase components) affect the size of the particles and the location of their phases. Vorobyova *et al.* obtained zinc oxide with different particle shapes (irregular aggregates of particles, needle shapes, near-spherical and near-hexagonal shapes, and spherical aggregates) and with diameters in the range: 2–10 μm, 90–600 nm, 100–230 nm and 150 nm respectively, depending on the process conditions.

An emulsion method was also used in the work of Lu and Yeh [59]. The aqueous phase of the system was zinc acetate dissolved in de-ionized water, and the organic phase was heptane. To stabilize the water-in-oil emulsion, the surfactant Span 80 was added to the heptane. NH_4_OH was added to the emulsion in order to obtain the zinc cation. The precipitate was dried, and then calcined at 700–1000 °C. The resulting ZnO calcinates were analyzed by XRD, IR and SEM. Lu and Yeh concluded that ZnO precipitated in this emulsion system has a smaller range of particle sizes (0.05–0.15 μm) compared with ZnO obtained in a traditional system (0.10–0.45 μm). The product consisted of nearly spherical particles.

**Figure 4.**
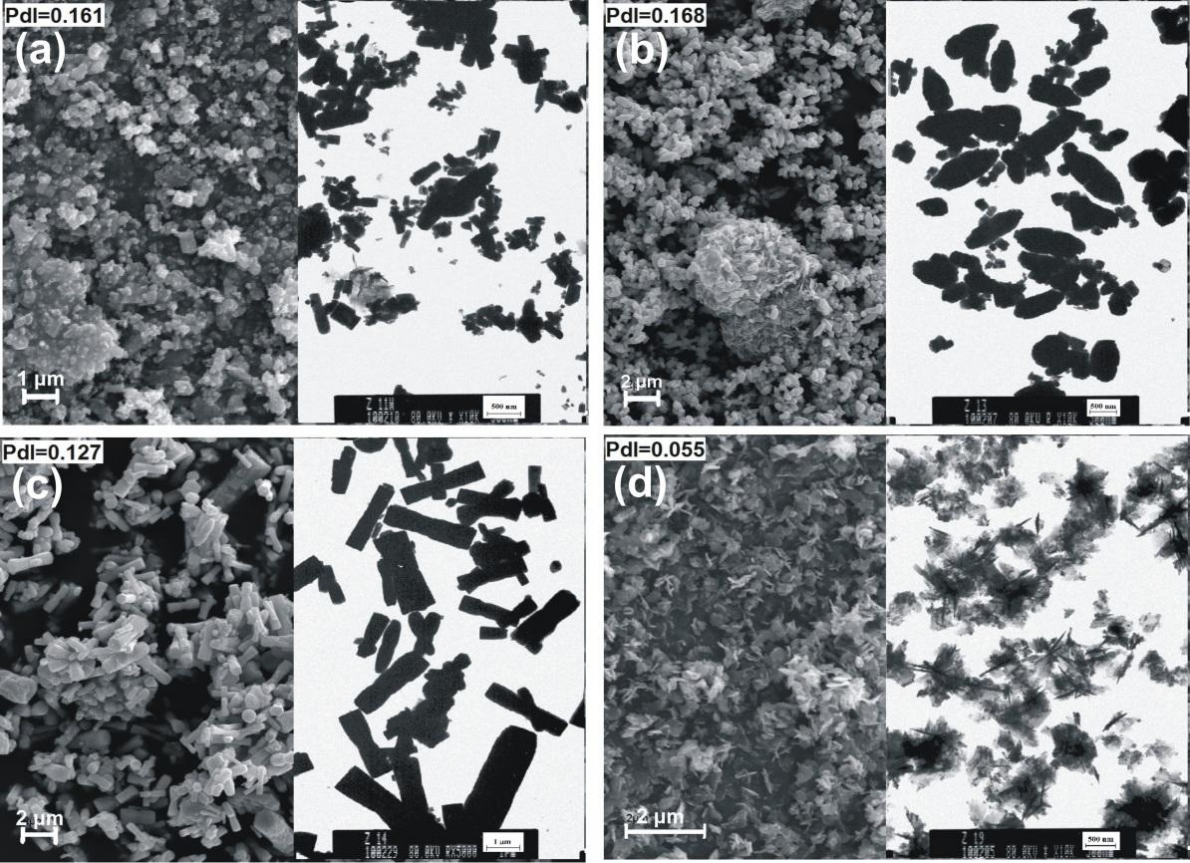
Zinc oxide structures: (**a**) solids; (**b**) ellipsoids; (**c**) rods; and (**d**) flakes [60].

Zinc oxide was also obtained by precipitation in an emulsion system with zinc acetate used as a precursor of ZnO, and potassium hydroxide or sodium hydroxide as precipitating agent [60]. Cyclohexane, as an organic phase, and a non-ionic surfactant mixture were also used for preparation of the emulsion. By applying modifications of the ZnO precipitation process, such as changing the precipitating agent, composition of substrates and the rate of substrate dosing, some interesting structures of ZnO particles were obtained. The morphology of the modified samples was analyzed based on SEM (scanning electron microscope) and TEM (transmission electron microscope) images. Moreover the samples were analyzed by determination of their dispersive properties using the non-invasive back scattering method (NIBS), parameters of porous structure (*BET*) and crystalline structure (XRD). Thermogravimetric analysis (TG) as well as infrared spectrophotometry (FTIR) were also applied. For selected samples their electrical properties (dielectric permittivity and electric conductivity) were also measured. The zinc oxide obtained consisted of particles in the shapes of solids, ellipsoids, rods and flakes (Figure 4), with sizes ranging from 164 to 2670 nm, and was found to have large surface area, with values as high as 20 m^2^/g.

Emulsions and microemulsions differ markedly from each other, which makes it relatively easy to identify the areas of their application. Microemulsions are stable, transparent, isotropic liquids consisting of an aqueous layer, and oil layer and a surfactant. The drop size in a microemulsion is significantly smaller than in an emulsion, and lies in the range 0.0015–0.15 μm [79–81]. In contrast to emulsions, microemulsions form spontaneously in appropriate conditions.

**Figure 5.**
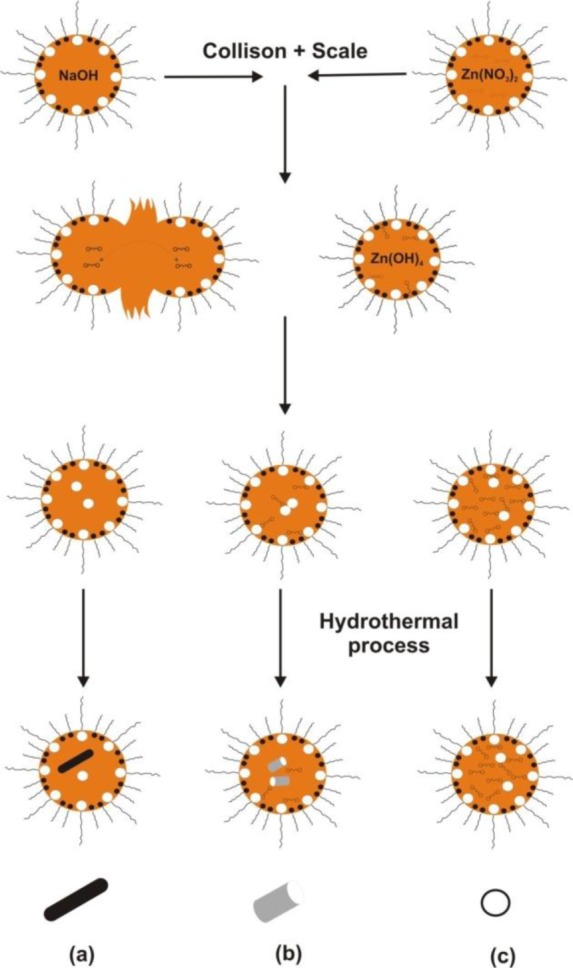
Synthesis and morphology of crystalline ZnO synthesized in a microemulsion system: (**a**) without PEG 400; and with the addition of: (**b**) 12.5%–25% PEG 400; (**c**) 50% PEG 400 (created based on [61] with permission from Elsevier Publisher).

Li *et al.* [61] proposed a method of preparing nanometric zinc oxide using a microemulsion which is formed when alcohol is added to an emulsion system consisting of water, oil and emulsifier, until a transparent mixture is obtained. In this case the microemulsion consists of a solution of heptane and hexanol together with a non-ionic surfactant (such as Triton X-100). The growth of nanoparticles involves the exchange of the substrates Zn(NO_3_)_2_ and NaOH between the microemulsion drops and the medium (poly(ethylene glycol)—PEG 400), and aggregation of the formed nuclei. Drops of microemulsion act as a microreactor in which the desired reaction takes place. In the synthesis of ZnO, different concentrations of PEG 400 were used (0%–50%). Figure 5 illustrates the process of synthesis in microemulsion and the shape of ZnO nanoparticles as proposed by the aforementioned authors.

Another process of precipitation of zinc oxide in the environment of a microemulsion was proposed by Singhal *et al.* [62]. Zinc oxide was obtained from a microemulsion consisting of ZnO-AOT/ethanol/isooctane (AOT—sodium bis-(2-ethylhexyl)-sulfosuccinate). For converting Na(DEHSS) (sodium diethylhexylsulfosuccinate) into Zn(DEHSS)_2_, an appropriate solvent was used, which dissolves Zn(DEHSS)_2_ but does not precipitate NaNO_3_. Appropriate quantities of Na(DEHSS) (dissolved in dry ether) and Zn(NO_3_)_2_ (dissolved in ethanol) were mixed for 4 h at room temperature. It is important that the solvents do not contain water, which might dissolve NaNO_3_ during the reaction, causing contamination of the precursor. The resulting solution was filtered and dried. Residues of water in the Zn(DEHSS)_2_ were removed by washing the precipitate with benzene. To ensure that the sample did not contain sodium, Zn(NO_3_)_2_ was also added to the solution. At the next stage an anhydrous microemulsion of alcohol in oil was prepared. For this purpose Zn(DEHSS)_2_ was dissolved in isooctane, and then dry ethanol was added. To the microemulsion prepared in this way, zinc oxalate was added in excess, in the form of a fine powder, so as to precipitate Zn^2+^ ions. The entire solution was mixed for 1 h. The precipitate was separated from the solution by centrifugation, and the precipitate obtained was then washed twice.

The first washing was performed using a methanol:chloroform mixture in a ratio of 1:1 by volume, to remove surfactant and oil. The second used an acetone:methanol mixture (1:1) to remove surfactant and excess oxalic acid. The dried precipitate was calcined at 300 °C for 3 h to produce nanoparticles of ZnO. Singhal *et al.* precipitated zinc oxide with particles in the range 11–13 μm and with a *BET* surface area of 82–91 m^2^/g, depending on the conditions applied.

The technique of obtaining ZnO using microemulsion was also used by Yildirim and Durucan [63]. They attempted to modify the microemulsion method so as to obtain monodisperse zinc oxide. They did not obtain zinc oxide directly from the microemulsion process, but used thermal decomposition of the zinc complex precipitated in the microemulsion process, followed by its calcination. The process was modified in that glycerol was used as the internal phase of a reverse microemulsion (Aeroloz OT:glycerol:heptane), similarly as Moleski *et al.* [64] did in preparing ZnO nanoparticles on amorphous silica. The basic aim of the work of Yildirim and Durucan was to determine how the concentration of surfactant and the temperature of calcination affect the size and morphology of the resulting ZnO particles. The final product was analyzed using such techniques as X-ray diffraction (XRD), field emission scanning electron microscopy (FESEM), thermogravimetry (TGA), differential scanning calorimetry (DSC), and luminescence spectroscopy (PL). The zinc oxide obtained consisted of spherical and monodisperse particles measuring 15–24 nm.

In turn, Xu *et al.* [37] compared three methods for precipitating zinc oxide: emulsion, microemulsion, and chemical precipitation. In all of these methods the starting solution used was zinc nitrate (Zn(NO_3_)_2_). The emulsion consisted of Zn(NO_3_)_2_ and an appropriate surfactant (cationic, anionic or non-ionic). However the microemulsion was prepared from Zn(NO_3_)_2_, cyclohexane, acrylonitrile-butadiene-styrene copolymer (ABS), butanol, and hydrogen peroxide (H_2_O_2_). All processes were carried out in a reactor at a temperature of 25 °C (for the chemical and emulsion processes) or 60 °C (for the microemulsion process). The precipitate was dried at 80 °C for 24 h, and then calcined at 600 °C for 2 h. On comparing the methods, the authors concluded that the smallest ZnO particles were obtained from the process carried out in a microemulsion environment (≤20 nm), larger ones were obtained from the emulsion (20–50 nm), and the largest were obtained by chemical precipitation (>50 nm). The size of the ZnO particles precipitated from the emulsion system depended on the type of surfactant: cationic (40–50 nm), non-ionic (20–50 nm) or anionic (~20 nm). In summing up their work, Xu *et al.* noted that the size of the ZnO particles depends on the method of precipitation and the type of surfactant used.

#### 2.2.6. Other Methods of Obtaining Zinc Oxide

There also exist many other methods of obtaining zinc oxide, including growing from a gas phase, a pyrolysis spray method, a sonochemical method, synthesis using microwaves, and many others.

Zinc oxide was obtained in the form of pure crystals by Grasza *et al.* [82]. Crystals of ZnO were grown from a gas phase (air, nitrogen, atmospheric oxygen, gaseous zinc and arsenic). A wide range of values of heating time and temperature were used. Particular emphasis was placed on analysing the surface during its interactions with the air, oxygen and gaseous zinc. The diversity of the morphology and the purity of the crystal surface were analyzed using AFM and XRD. It was found that thermal heating in the various gases led to similar changes in the crystal surface, although differences were observed in the rate of those changes. Grasza *et al.*, showed that heating in gaseous zinc leads to a surface roughness of less than 1 nm, while heating in gaseous arsenic causes degradation of the crystal surface. Tests showed that the porosity of the crystal surface increases with increasing temperature and heating time. The “milky” crystal surface obtained is a result of imperfections arising during the preparation of the surface for heating.

A thin layer of zinc oxide was obtained by Wei *et al.* [83] in an atmosphere of O_2_, under a pressure of 1.3 Pa, using the pulsed laser deposition method (PLD), with powdered and ceramic ZnO. They determined the effect of temperature on the structural and optical properties of the thin ZnO layer, using techniques such as XRD, SEM, FTIR and PL spectra. The results obtained by Wei *et al.* indicate that the best structural and optical properties of ZnO are obtained for a thin layer of zinc oxide produced at 700 °C using ZnO powder, and at 400 °C using ceramic ZnO. The PL spectra indicate that UV emission increases with increasing temperature. It was found that the quantity of UV emitted for a thin layer of ZnO made using powder was smaller than in the case of ceramic ZnO.

Using an aerosol pyrolysis method, Zhao *et al.* [65] obtained ultrapure particles of ZnO. As a zinc precursor they used Zn(CH_3_COO)_2_·2H_2_O, in view of its high solubility and low temperature of decomposition. A determination was made of the mechanism and kinetics of the thermal decomposition of zinc acetate dihydrate, as well as a correlation of the mechanism with the results of the aerosol pyrolysis process. Analysis of the DTA and TG curves shows that the water of crystallization is lost below 200 °C, and anhydrous zinc acetate begins to form. At 210–250 °C the anhydrous zinc acetate decomposes into ZnO and organic compounds by way of endothermic and exothermic reactions. The process of decomposition of Zn(CH_3_COO)_2_·2H_2_O is complete at 400 °C. Zinc oxide synthesized by the aerosol pyrolysis method consists of particles in the range 20–30 nm.

Hu *et al.* [57] produced connected rods of ZnO using a sonochemical process (exposure to ultrasound in ambient conditions), and by microwave heating. In the sonochemical method an aqueous solution of Zn(NO_3_)_2_·6H_2_O was added to (CH_2_)_6_N_4_ (hexamethylenetetramine, HMT). The resulting solution was exposed to ultrasound for 30 min, as a result of which the reaction temperature reached approximately 80 °C. In the second method, the solution was heated using microwaves at 90 °C for 2 min. The precipitates obtained were centrifuged, washed with water and ethanol, and dried at 60 °C for 2 h. The resulting ZnO was analyzed using XRD, TEM, SAED (selected area electron diffraction), EDS, and PL spectra. These methods of obtaining ZnO enable high yields, in excess of 90%. In summing up their work, Hu *et al.* stated that the sonochemical and microwave heating methods do not require surfactants, can be applied on a large scale with low production costs, and are simple and energy-efficient. The ZnO rods obtained can be successfully used in electronics and optoelectronics. The sonochemical method may be used in the future for the synthesis of single-dimensional structures of other metal oxides.

## 3. Methods of Modification of Zinc Oxide

**Figure 6.**
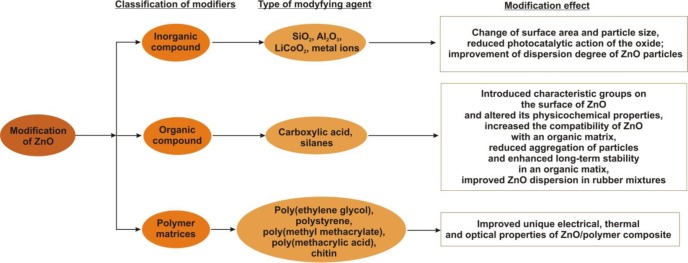
Schematic diagram of the most popular modifying methods of ZnO.

The search for new possible applications of zinc oxide, the need to reduce its content in rubber mixtures, and the major problem of its tendency to form significant agglomerations have encouraged researchers in recent years to carry out numerous studies to find an optimum method of modifying the surface of the compound without impairing its physicochemical properties. Modification is also often carried out in order to improve its performance properties, such as high or low (depending on application) photocatalytic activity. In the following sections we will consider the methods of modification of zinc oxide proposed by various scientists. Figure 6 presents a schematic that summarizes all the method of modifiction of ZnO mentioned in the text.

**Figure 7.**
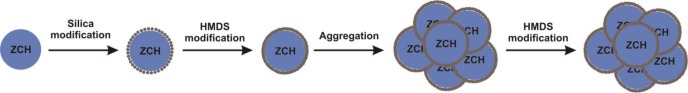
Schematic representation of the synthesis of surface-modified ZnO ultrafine particles using an *in situ* modification method (created based on [41] with permission from Elsevier Publisher).

Cao *et al.* [41] performed modification of zinc oxide using silica and trimethyl siloxane (TMS). The finest particles of ZnO were obtained by calcination of the precursor zinc carbonate hydroxide (ZCH). ZHC was obtained in a process of precipitation from substrates such as zinc sulfate heptahydrate (ZnSO_4_·7H_2_O), ammonium solution (NH_4_OH) and ammonium bicarbonate (NH_4_HCO_3_). The surface of the ZCH was then successively modified by an *in situ* method using TEOS and hexamethyldisilazane (HMDS) in water. The ZHC functionalized in this way was calcined, to obtain ultrafine particles of ZnO. Modification of the ZnO particles made possible a solution to the problem of their agglomeration. Functionalization of the ZnO surface with an inorganic compound (silica) reduced the photocatalytic action of the oxide, while the organic compound (HMDS) increased the compatibility of the ZnO with an organic matrix. The highly transparent modified zinc oxide surface was found to provide excellent protection against UV radiation, which represents a significant advantage of the use of these modifying agents. A schematic representation of the synthesis of surface-modified ZnO ultrafine particles using an *in situ* modification method is shown in Figure 7.

Modification with the use of silica was also performed by Xia and Tang [84]. By a method of controlled precipitation, clusters of zinc oxide were obtained on the surface of silica modified using triethanolamine N(CH_2_CH_2_OH)_3_ (TEOH) and containing silanol (≡Si-OH) and siloxane (≡Si-O-Si≡) groups. Molecules of TEOH are adsorbed by the silica, and the siloxane and silanol networks are broken as a result of the changes occurring in the SiO_2_. The Zn^2+^ ions, in reaction with triethanolamine, produce clusters of ZnO on the silica surface. In accordance with the theory of maturing and aggregation, the resulting clusters are susceptible to rapid collision with other clusters of zinc oxide, leading to an appropriate concentration of the compound. An important role in the proposed modification technique is played by TEOH, which enables complex structures to be obtained.

Hong *et al.* [36] also performed modification of zinc oxide using silica. They also performed an additional modification using oleic acid. Zinc oxide was obtained as a result of the reaction of zinc acetate with ammonium carbonate, followed by calcination of the resulting zinc precursor. To determine the compatibility between the inorganic nanoparticles and the organic matrix, the surface of the ZnO was covered with oleic acid. The FTIR spectra confirmed the presence on the surface of the modified ZnO of an organic layer and a chemical bond between the inorganic –OH groups and the organic chain macromolecules. The proposed mechanism for these processes was presented in terms of reaction (16):

$$\text{ZnO}{\left(\text{OH}\right)}_{x}+y\text{HOC}{\left({\text{CH}}_{2}\right)}_{7}\text{CH}=\text{CH}{\left({\text{CH}}_{2}\right)}_{7}{\text{CH}}_{3}\to \text{ZnO}{\left(\text{OH}\right)}_{x-y}{\left[\text{OOC}{\left({\text{CH}}_{2}\right)}_{7}{\text{CH}}_{3}\right]}_{y}+yH_{2}O$$ (16)

When SiO_2_ was used as a modifier, the FTIR spectrum indicates the presence of an interphase bond between ZnO and SiO_2_. Coverage of the zinc oxide surface with a thin film of amorphous silica improved the degree of dispersion, and thus reduced the agglomeration of nanoparticles. Moreover, based on photocatalytic degradation in aqueous solution using methyl orange, it was shown that silica-coated ZnO has lower catalytic activity than the original nanostructures. The work of Hong *et al.* showed that heterogeneous azeotropic distillation of the zinc precursor completely reduces the crystalline structure of ZnO, and thus makes it possible to avoid large aggregation and reduces the average particle size. Similar studies have been carried out and published by those authors in [85].

Hydrophobic ZnO nanoparticles were produced by Chen *et al.* [86] and a novel treatment process was developed by them to obtain highly dispersed and long-term stable ZnO nanoparticles, in an organic matrix. Aminopropyltriethoxysilane (APS) was grafted onto the surface of ZnO nanoparticles, and a long carbon chain of stearic acid (SA) was introduced through a condensation reaction between APS and the activated SA with *N*,*N′*-carbonyldiimidazole (CDI). ZnO nanoparticles were analyzed by FTIR, TGA, SEM and a sedimentation test. The FTIR and TGA results showed that APS and SA were linked on the surface of ZnO nanoparticles through chemical bonds, and the CDI activator clearly promotes the condensation reaction and increases the grafting ratio of SA. Results from the SEM observations and sedimentation test indicate that the new surface treatment would considerably reduce aggregates of particles and enhance long-term stability in an organic matrix.

Modification of ZnO using an inorganic compound, namely Al_2_O_3_, was carried out by Yuan *et al.* [87]. Nanometric zinc oxide coated with Al_2_O_3_, with diameter 50–80 nm, was obtained by calcination of basic zinc carbonate (BZC) with simultaneous modification with a precipitate of Al(OH)_3_ at 400–600 °C. The coating obtained was highly uniform, and had a thickness of 5 nm. The pH at the isoelectric point for ZnO nanoparticles with an Al_2_O_3_ layer moved from around 10 to a value of 6, which may improve the dispersion of ZnO particles.

Wysokowski *et al.* [88] decided to develop a ZnO-containing composite material using β-chitin from *Sepia officinalis* cephalopod mollusk as the source of chitin. They suggest that application of morphologically defined β-chitin as a template for biomimetic ZnO deposition is very attractive from a technological point of view as it eliminates challenges associated with manufacturing chitin to chitosan and with processing to membranes or scaffolds.

Pyskło *et al.* [89] performed modification of zinc oxide using poly(ethylene glycol) and octadecyltrimethoxysilane, in order to improve its dispersion in rubber mixtures. The modification used zinc oxide synthesized by a hydrothermal method (microwave dehydration). The modification was carried out in the following way: in a solution containing 5% by mass of modifier (PEG or silane) relative to the mass of ZnO used, a ZnO nanopowder was dispersed. The resulting system was then mixed using an ultrasound disintegrator (in the case of silane the system was first mixed with a magnetic mixer, with simultaneous heating). The resulting precipitate was filtered and dried at a temperature of 80 °C for 48 h. Analysis of the FTIR spectra revealed the presence of -OH groups on the surface of the ZnO; for this reason the ZnO surface was modified with octadecyltrimethoxysilane. The modification was carried out as follows: ZnO was dispersed in an emulsion containing 5% by weight of silane. The whole was mixed with a magnetic mixer, with heating, for 5 min. The resulting precipitate was filtered and dried for 48 h at 80 °C. The addition to rubber mixtures of PEG-coated nano zinc oxide an increase in the degree of cross-linking of the vulcanizates, but there was also an increase in vulcanization reversion and a marked decrease in prevulcanization time. The samples of ZnO obtained were additionally analyzed using inverse gas chromatography (IGC), in order to determine the dispersive component of surface energy (γsD). Based on the results it was concluded that coating the surface of nanopowders with polyglycol or silane causes a decrease in the value of γsD (a better effect was obtained when PEG was used). The marked decrease in surface energy in the case of oxides modified with PEG and silane can be expected to facilitate their dispersion in nonpolar rubbers.

Modification of the surface of ZnO particles using silane was also performed by Kotecha *et al.* [90]. The modifier used was 3-methacryloxypropyltrimethoxysilane. Nanoparticles of zinc oxide were obtained using zinc acetate and potassium hydroxide as substrates. The precipitate was filtered and washed with methanol, and then dried at 130 °C. In this method the silane was introduced into the system during the precipitation. Concurrently with the formation of ZnO particles, a reaction takes place between silane and ZnO. In the course of this reaction H_2_O is generated and a side reaction takes place, during which the pH increases to 9. The silane-covered zinc oxide particles were introduced into an aqueous suspension and exposed to UV radiation. Based on interpretation of SEM images, the researchers concluded that unmodified zinc oxide contains particles around 100 nm in diameter, forming agglomerates. The introduction of silane into the ZnO structure caused a decrease in the particle size (40–100 nm) and an increase in the diameters of the aggregates, even to the order of micrometres. The irradiated ZnO particles had a fibrous structure “resembling wool”, and offered promising catalytic properties. UV radiation also changes the character of ZnO from hydrophobic to hydrophilic. Analysing the adsorption parameters, Kotecha *et al.* found that the surface area of silane-modified ZnO initially increases together with the concentration of silane, until that concentration reaches a value of approximately 1–2 mol—then the *BET* surface area starts to decrease. For the irradiated ZnO samples, the value of *BET* surface area continues to increase as the silane concentration increases, reaching a maximum of approximately 130 m^2^/g for the highest concentration. The results of Kotecha *et al.* imply that UV irradiation destroys organic domains. The resulting material has high porosity, large *BET* surface area, and hydrophilic properties.

**Figure 8.**
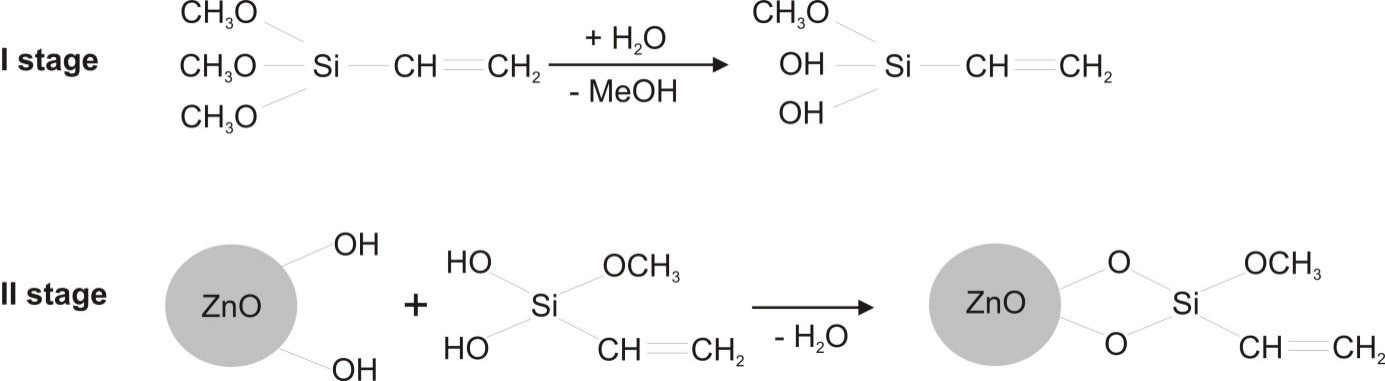
Probable mechanism occurring during modification of ZnO using vinyltrimethoxysilane.

Figure 8 shows example mechanisms taking place during the process of modification of zinc oxide using a selected silanol binding compound.

Chang *et al.* [91] modified the surface of ZnO using LiCoO_2_. Zinc oxide covered with a layer of LiCoO_2_ was obtained by plasma-enhanced chemical vapour deposition (PE-CVD). In their work, Chang *et al.* confirmed the favourable effect of ZnO on the electrochemical yield and thermal stability of LiCoO_2_, which is used as a cathode material in Li-ion batteries. Covering ZnO with a layer of LiCoO_2_ causes an increase in the surface area of the functionalized ZnO (from 0.4 to 1 m^2^/g).

Change of state of the surface of zinc oxide nanowires through plasma treatment is one of the most promising methods of ZnO modification. Experiments carried out by Ra *et al.* [92] aimed to determine how reactive chemical treatment using oxygen affects electrical transport, gas selectivity and the internal photoelectric effect of ZnO nanowires with a diameter of 80 nm, using a field-effect transistor (FET). A significant increase in the concentration of oxygen was observed, in the form of active oxygen centres (O^2−^ and OH^−^) on the nanowire surface. After treatment the concentration of the carrier and mobility of the ZnO decreased. There was also an improvement in properties relating to the detection of hydrogen by the modified nanowires, and the time of photocurrent amplification in UV radiation. In summarizing their work, Ra *et al.*, stated that modification of the surface of ZnO using plasma treatment with oxygen opens up new possibilities for the production of electronic devices, catalysts and high-performance sensors.

In turn, Kang and Park [93] modified zinc oxide using silver ions. The ZnO was prepared using ultrasonic aerosol pyrolysis (FEAG) of a colloidal solution of zinc acetate. The size of the ZnO particles obtained by the FEAG method depended on the conditions of the operation and the type of solvent. Based on TEM images it was found that the ZnO obtained consisted of particles measuring approximately 12 nm. Next the ZnO was dispersed in a solution of silver nitrate in various ratios. As the ZnO:Ag mass ratio was increased, there was a change in the product’s surface area and particle size. Kang and Park obtained a ZnO-Ag composite with particles measuring approximately 120–250 nm and with a surface area of 3–6 m^2^/g.

Šćepanović *et al.* [94] modified ZnO using mechanical activation. A commercial ZnO powder was activated mechanically by grinding in a vibrating mill with steel rings, under continuous air circulation. The process was continued for 30 and 300 min. The product was subjected to comprehensive physicochemical analysis. Based on SEM images, Šćepanović *et al.* noted that the size of the crystallites of the modified ZnO was smaller, and the surface area was larger, than in the case of the unmodified product (for example, from 190 nm to 106 and 44 nm, and A_BET_ from *ca.* 3 m^2^/g to 4 and 6 m^2^/g).

Wu *et al.* [95] produced ordered ZnO nanofibres by an electrospinning method, and modified the nanofibres using CdS with a nanocrystal layer deposition method. They then investigated the performance of hybrid solar cells based on the CdS/ZnO nanofibres and P3HT (poly(3-hexylthiophene)). The devices were optimized by changing the number of layers of cross-aligned ZnO nanofibres and the growth time of CdS on the ZnO. Wu *et al.*, found that the power conversion efficiency (PCE) of such a hybrid solar cell was improved by more than 100% after CdS modification. In addition, the lifetime of carriers at the bulk heterojunction was investigated using an impedance analyser and was found to be dramatically increased after CdS modification.

Over the past decade much work has been done on developing nanocomposites produced by the action of modified inorganic carriers with polymer matrices. Such procedures make it possible to produce new classes of polymeric materials which combine properties of both inorganic particles and organic polymer matrices (including process ability and elasticity). The MO/polymer composites produced in this way have unique electrical, thermal and optical properties, which enable their range of applications to be extended in many branches of industry [96–101].

Shim *et al.* [102] carried out modification of zinc oxide using poly(methyl methacrylate) (PMMA). A ZnO/PMMA composite was synthesized by means of polymerization *in situ*. The majority of microspheres of the MO/polymer composite are produced by coupling of existing polymer chains with the inorganic surface or by polymerization on the phase boundary of inorganic particles. Shim *et al.*, demonstrated that the stability of dispersion of ZnO in a monomer depends strongly on the nature of its surface, since this provides a precondition enabling dispersion of particles of the medium within drops of monomer and consequently their enclosure in PMMA. The most important condition in the production of the composite is the interphase compatibility between the inorganic compound and the polymer. For this purpose the surface of the inorganic system should be treated with a hydrophobic organic substance. The obtained inorganic-polymer composites form persistent microspheres and combine easily into highly processed polymers. Similar studies have been carried out and published by other researchers [103,104].

Tang *et al.* [105] modified zinc oxide using poly(methacrylic acid) (PMAA). The hydroxyl groups on the ZnO surface reacted with the carboxyl groups of the PMAA, producing a complex of poly(zinc methacrylate) on the surface of the zinc oxide. Interpreting the particle size distributions, it was found that the ZnO modified with PMAA contains particles with smaller diameter (*ca.* 70 nm) compared with unmodified ZnO (*ca.* 300 nm). Analysis of the dispersive stability of the ZnO showed that the modified particles of zinc oxide dispersed better in water than unmodified particles. Conventional inorganic nanoparticles have hydroxyl groups (-OH) on their surface, due to the effect of humidity and the environment and type of precipitation. These groups react with COO- groups to form small complexes of poly(zinc methacrylate) on the surface of the zinc oxide. Analysis using the FTIR, TGA, TEM and XRD techniques confirms the presence of polymer molecules on the zinc oxide surface.

Poly(methyl methacrylate) was also used as a ZnO surface modifier by Hong *et al.* [106]. Nanoparticles of zinc oxide with a diameter of approximately 30 nm were synthesized by means of homogeneous precipitation followed by calcination. In order to introduce reactive groups onto the ZnO surface, a reaction was carried out between the hydroxyl groups and a silane coupling agent (3-methacryloxypropyltrimethoxysilane). Graft polymerization was effected by means of a reaction between the ZnO, containing silanol groups, and the monomer. Tests showed that the polymerization does not alter the crystalline structure of the ZnO nanoparticles. Their dispersion in the organic solvent can greatly improve the graft polymerization of PMMA, and further improvement can be achieved by the addition of other surfactants. Modification of ZnO nanoparticles by grafted PMMA increases the degree of lyophilicity of the inorganic surface and reduces the formation of aggregates. The work of Hong *et al.*, showed that ZnO nanoparticles grafted with PMMA can increase the thermal stability of polystyrene.

Polystyrene (PS) is also the subject of interest as a surface modifier of zinc oxide particles. Chae and Kim [107] carried out a process of ZnO surface modification using that compound. In the process of obtaining a PS/ZnO composite, first an appropriate quantity of commercial ZnO (particle size 87 nm) was dispersed in a solvent with the help of ultrasound for 10 min. The solvent used was *N,N*-dimethylacetoacetamide (DMAc). Next, in the resulting DMAc/ZnO solution, polystyrene was dissolved, mixing vigorously for 2 h at 70 °C. To obtain a layer of nanocomposite the solutions were kept at a temperature of 90 °C for 4 days. Then the layer was dried (at 100 °C for 5 days) and hot pressed (at 200 °C), completely removing the remaining DMAc. For the structures obtained, the morphology, microstructure, thermal properties and mechanical properties were investigated. Spectral and X-ray identification were also performed, using techniques including TEM, FESEM, DSC, TGA, FTIR and WAXS. The tests confirmed that the solvent used is capable of breaking up the agglomerates that form, and prevents re-agglomeration during mixing of the solution.

An object of interest in recent years has been the resistance connections of ZnO particles embedded in MIM (metal-insulator-metal) structures. Work has focused on altering the layers of oxides, whose amorphous nature, porosity and lack of homogeneity constitute a problem. Researchers under the direction of Verbakel [108] investigated the resistance effects of the switching of diodes containing structures of nanometric ZnO covered with an active layer from a polystyrene matrix. These diodes consist of two PEDOT:PSS electrodes. Using an impedance spectroscope it was found that the electronic memory effect in nanostructured metal oxides can be affected by modification of the surface of the particles using coordinating ligands (e.g., amines and thiols), and this depends on the temperature of voltage measurements. This process provides new prospects for ecological modification of the surface of ZnO powder using inorganic hybrid materials.

**Figure 9.**
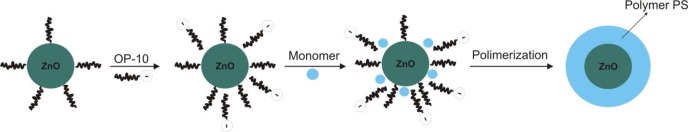
Mechanism of nano-ZnO/PS composite synthesis by *in situ* emulsion polymerization (created based on [109] with permission from Elsevier Publisher).

Modification of ZnO nanoparticles using polystyrene was also performed by researchers under the direction of Tang [109]. Nanometric particles of zinc oxide (particle size *ca.* 40 nm) were “enclosed” in polystyrene, with a process of emulsion polymerization being carried out *in situ* in the presence of 3-mercaptopropyltrimethoxysilane (MPTMS) as a coupling agent and polyoxyethylene nonylphenyl ether (OP-10) as surfactant. The nano-ZnO surface had to have a hydrophobic character, in order to hermetically seal the ZnO nanoparticles perfectly in the monomer. This property was controlled by the creation of functional groups on the nano-ZnO surface with the use of a silane coupling agent (MPTMS). Consequently the MPTMS molecules were grafted on the surface of the nano-ZnO. MPTMS is an organic polymer chain which forms steric hindrances between inorganic particles, preventing their aggregation. However it was not simple to obtain perfect dispersion of the hydrophobic nano-ZnO particles in an aqueous polymerization system. To ensure stability of dispersion, a surfactant (OP-10) was added to the system, in a quantity smaller than that which properly saturates the surface, so as to avoid the formation of micelles of emulsifier. Tang *et al.*, proposed a mechanism for the polymerization (Figure 9). Tests showed that the particles of the resulting polymer composite are monodisperse, with diameters in the range 150–250 nm.

Another modifier applied on the surface of ZnO is polyacrylnitryl (PAN). Studies with that compound were carried out by Chae and Kim [110]. ZnO nanopowder (particles of diameter 87 nm) was dissolved in DMAc to break up agglomerates. PAN was then added to the solution, and it was mixed vigorously at 70 °C. To obtain the nanocomposite, the PAN-ZnO solution was kept at 80 °C for 4 days, and at the next stage was dried at 100 °C for 5 days. The resulting precipitate underwent spectroscopic, thermal and mechanical analysis. The product exhibited better thermal stability than the starting material, due to the barrier role of ZnO. Moreover the ZnO nanoparticles caused a reduction in the crystallization temperature of the modifier (PAN) and an increase in the width of the crystallization peaks. This is linked to heterogeneous nucleation and the reduced mobility of the polymer chains. The introduction of ZnO nanoparticles into the polymer chain caused an increase in the modulus of elasticity on stretching and a reduction in the dynamic load resistance.

Xiong *et al.* [111] synthesized a new nanocomposite ZnO(PEGME), in which the ZnO nanoparticles and polymer groups (PEGME—poly(ethylene glycol) methyl ether) are linked by covalent bonds. The compound was analyzed in terms of composition, structure, fluorescence and specific conductance. The tests showed that the polymer nanocomposite synthesized by means of a chemical reaction has better properties than its equivalent obtained through physical mixing. The lasting stability of the properties of ZnO(PEGME) results from the strong chemical bond between the polymer and the nanoparticles. The hybrid ZnO(PEGME) has the capability of tuning luminescence spectra and has stable ionic conductance. These properties mean that the obtained compound can be used in luminescent devices and in electronic apparatus.

**Figure 10.**
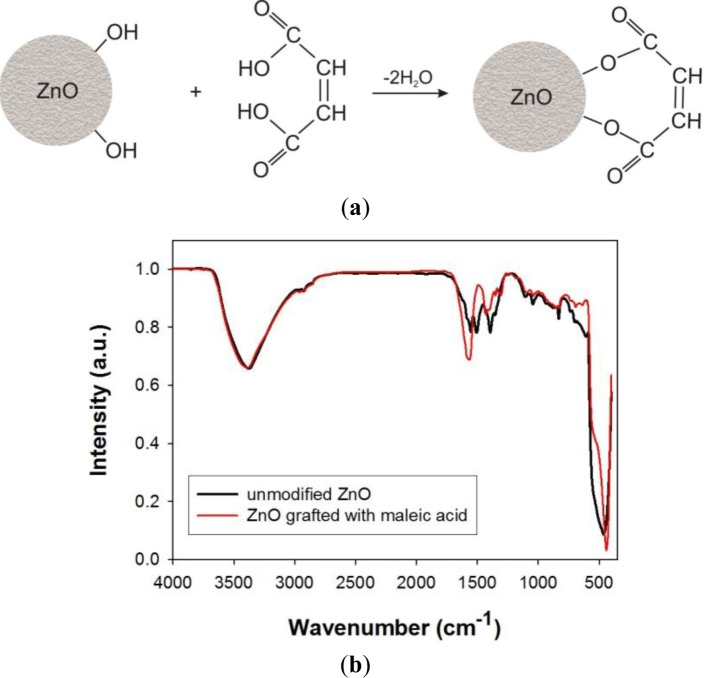
(**a**) Probable mechanism of ZnO surface modification with maleic acid; and (**b**) exemplary FTIR spectra of obtained products.

Modification of zinc oxide using carboxylic acids (such as stearic, tartaric, maleic, propanoic *etc.*) makes it possible to introduce characteristic groups onto the surface of the ZnO and to alter its physicochemical properties. Studies of zinc oxide modified with carboxylic acids (wet modification) have shown that they do not significantly affect the morphological/dispersive or porous properties of zinc oxide. An apparently promising method is modification *in situ*, which causes a significant increase in the surface area of the zinc oxide (to as high as *ca.* 30 m^2^/g). Figure 10 shows an example mechanism taking place during modification of zinc oxide with maleic acid, and an FTIR spectrum confirming the effectiveness of the modification.

It has been experimentally demonstrated that alkanethiols may adsorb on ZnO surface [112–114]. For example, Singh *et al.* [113] have investigated adsorption, in ultrahigh vacuum, of methanethiol (MT), 1-dodecanethiol (DDT) and 3-mercaptopropyltrimetoxysilane (MPTMS) on sputter-cleaned ZnO(0001) via either the silane or thiol and of the molecule. They also presented the first ultraviolet photoelectron spectroscopy (UPS) investigation of thiol adsorption on zinc oxide. It was found that the MT frontier orbitals are strongly perturbed by adsorption on ZnO(0001), with the work function of the surface increasing by 0.7 eV. X-ray photoelectron spectroscopy (XPS) and Raman spectroscopies confirmed adsorption, and *in situ* photoluminescence measurements showed the intensity of the visible emission peak is decreased by methanethiol adsorption. Their other work [114] demonstrates a previously unreported method of encapsulating zinc oxide nanoparticles and nanorods within an organic matrix consisting of a 1:2 Zn/thiol complex. The thickness and morphology of the encapsulating layer was controllable by the choice of thiol and preparation conditions. Singh *et al.*, concluded that this method may be useful in future photovoltaic applications in which one wishes to surround ZnO nanorods and whiskers with light-absorbing molecules, which could be achieved by using thiol-terminated dye molecules.

## 4. Applications of Zinc Oxide

**Figure 11.**
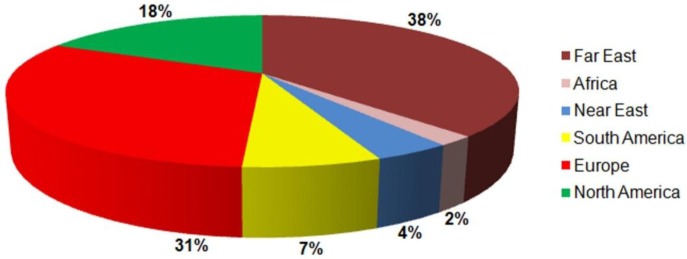
Worldwide consumption of zinc oxide.

Because of its diverse properties, both chemical and physical, zinc oxide is widely used in many areas. It plays an important role in a very wide range of applications, ranging from tyres to ceramics, from pharmaceuticals to agriculture, and from paints to chemicals. Figure 11 shows worldwide consumption of zinc oxide by region.

**Figure 12.**
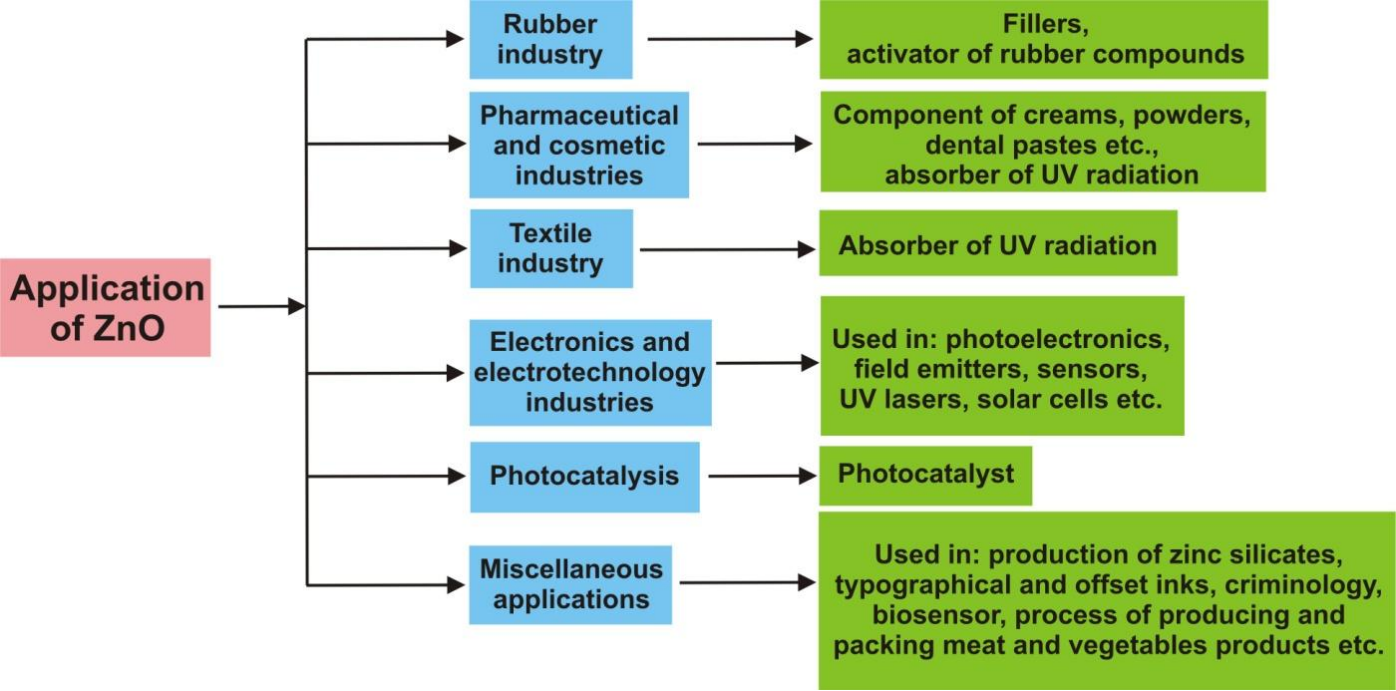
Schematic representation all the application of ZnO mentioned in the text.

In the Figure 12 summarized application paths of ZnO are presented.

### 4.1. Rubber Industry

Global production of zinc oxide amounts to about 10^5^ tons per year, and a major portion is consumed by the rubber industry to manufacture various different cross-linked rubber products [115]. The thermal conductivity of typical pure silicone rubber is relatively low; however, it can be improved by adding certain thermal conductivity fillers, including metal powders, metal oxides and inorganic particles. Some kinds of thermal conductivity powder, such as Al_2_O_3_, MgO, Al_2_N_3_, SiO_2_, ZnO, *etc.*, can improve the thermal conductivity of silicone rubber while retaining its high electrical resistance, and are thus promising candidates as high-performance engineering materials. The incorporation of nano-scale fillers can achieve high thermal conductivity even at a relatively low filling content. However, the ZnO nanoparticles tend to aggregate together to form particles of large size in the polymer matrix, due to the weak interaction between the surface of the nanoparticles and the polymer.

In order to solve this problem, surface modification techniques are applied to improve the interaction between the nanoparticles and the polymer. In the work of Yuan *et al.* [116], in order to prepare the silicone rubber with high thermal conductivity, pristine and surface-modified ZnO nanoparticles containing the vinyl silane group are incorporated into the silicone rubber via a hydrosilylation reaction during the curing process. The corresponding structure, morphology and properties of the silicone rubber/ZnO (SR/ZnO) and silicone rubber/SiVi@ZnO (SR/SiVi@ZnO) nanocomposites were investigated. Yuan *et al.* synthesized ZnO nanoparticles (with an average size below 10 nm) by a sol-gel procedure. Next the silicone coupling agent VTES was successfully incorporated onto the surface of the nanoparticles. The SR/SiVi@ZnO nanocomposites showed better mechanical properties and higher thermal conductivity due to the formation of a cross-linking structure with the silicone rubber matrix and better dispersion in that matrix.

Zinc oxide is a very effective and commonly used cross linking agent for carboxylated elastomers [117,118]. It can be used to produce vulcanizates with high tensile strength, tear resistance, hardness and hysteresis. The improved mechanical properties of ionic elastomers mainly result from their high capacity for stress relaxation, due to elastomer chain slippage on the ionic cluster surface and reformation of ionic bonds upon external deformation of the sample. Moreover, ionic elastomers have thermoplastic properties and can be processed in a molten state as a thermoplastic polymer [119]. However, there are some disadvantages to zinc-oxide-cross linked carboxylic elastomers. The most important are their scorchiness, poor flex properties and high compression set. In order to prevent scorchiness, carboxylated nitrile elastomers are cross linked with zinc peroxide or zinc peroxide/zinc oxide systems. The vulcanization of XNBR with zinc peroxide mainly leads to the formation of ionic crosslinks; covalent links are also formed between elastomer chains due to the peroxide action. However, higher vulcanization times are required to achieve vulcanizates with a tensile strength and crosslink density comparable to that of vulcanizates cross linked with zinc oxide. In the case of XNBR vulcanization with zinc peroxide/zinc oxide systems, curing is the sum of at least three processes: the very fast formation of ionic crosslinks due to the initial zinc oxide present, peroxide cross linking leading to the formation of covalent links (peroxide action), and ionic cross linking due to the production of zinc oxide from peroxide decomposition. The last process, which decays with vulcanization time, most likely involves the formation of ionic species. The achieved vulcanization times are considerably higher than in the case of XNBR cross linking with zinc oxide. Therefore, apart from the scorch problems, zinc oxide is still commonly used as a cross linking agent in carboxylated nitrile rubbers.

In view of the fact that, during the cross linking process, zinc oxide reacts with the carboxylic groups of the elastomer, which leads to the formation of carboxylic salts (ionic crosslinks), the most important parameters influencing the activity of zinc oxide are its surface area, particle size, and morphology. These parameters determine the size of the interphase between the cross linking agent and elastomer chains [120].

Przybyszewska *et al.* [121] used zinc oxides with different surface areas, particle sizes and morphologies (spheres, whiskers, snowflakes) as cross linking agents of carboxylated nitrile elastomer, in order to determine the relationship between the characteristics of zinc oxide and its activity in the cross linking process. They concluded that the use of zinc oxide nanoparticles produced vulcanizates with considerably better mechanical properties and higher crosslink density, as compared with vulcanizates cross linked with micro-sized zinc oxide, which is used commercially as a cross linking agent. Vulcanizates containing the same quantity of zinc oxide nanoparticles exhibited a tensile strength about four times greater than that of vulcanizates with micro-sized particles. Therefore the use of nano-sized zinc oxide enables the quantity of zinc oxide to be reduced by almost 40%. This is a very important ecological goal, since zinc oxide is classified as toxic to aquatic species, and the European Union requires that the amount of zinc oxide in rubber compounds be reduced. Moreover, it should be noted that vulcanizates of carboxylated nitrile elastomer cross linked with zinc oxide demonstrate heat shrinkability.

It is the morphology of zinc oxide particles which mainly affects the activity in the cross linking process. Particle size and surface area do not seem to have a significant influence on the efficiency of zinc oxide as a cross linking agent. The highest activity was observed for zinc oxide with a surface area of about 24 m^2^/g and three-dimensional snowflake particles. The specific shape and complex structure of ZnO aggregates, consisting of wires or plates growing from a single core, provide an increase in the size of the interphase between the elastomer carboxylic groups and the snowflake particle. As a result, Przybyszewska *et al.*, obtained vulcanizates exhibiting the best mechanical properties, mainly due to the high content of ionic clusters, which create multifunctional and labile crosslinks and can rearrange upon external stress, leading to stress relaxation. Moreover, the zinc oxide nanoparticles used by Przybyszewska *et al.*, have the lowest propensity for agglomeration in the elastomer matrix and create the smallest agglomerates, which concentrate the stresses during sample deformation to a smaller degree than the large agglomerates formed by other zinc oxides.

As mentioned above, the substantial usage of zinc oxide in rubber products has raised questions about the environmental impact of the rubber industry, particularly when this compound in finally released into the lithosphere during degradation of the rubber, after the end of a product’s life [122]. Environmental concerns are especially focussed on the effect of excess zinc on aquatic organisms [123], which has led to various efforts to reduce zinc levels in rubber compounds [124]. There are three basic methods of reducing the content of ZnO in rubber compounds:

(i) replacing the commonly used zinc oxide of diameter 0.1–0.9 μm and surface area 4–10 m^2^/g with active zinc oxide of nanoscopic granularity and surface area of up to 40 m^2^/g;
(ii) modifying the surface of the zinc oxide with carboxylic acids (such as stearic acid, maleic acid and others);
(iii) using additional activators [69].

To find an alternative to conventional ZnO, which in higher dosage is toxic to aquatic systems, Thomas *et al.* [125] synthesized the novel accelerators *N*-benzylimine aminothioformamide(BIAT)-capped-stearic acid-coated nano-ZnO (ZOBS), BIAT-capped ZnO (ZOB), and stearic acid-coated nano zinc phosphate (ZPS), to investigate their effects in NR vulcanization. They studied the effect of these capped compounds on the curing and mechanical properties of natural rubber (NR) vulcanizates. The zinc oxide used in the research was prepared by a sol-gel method, and was then modified using accelerators such as BIAT and fatty acids such as stearic acid. This capping technique reduces agglomeration of nanoparticles of ZnO and is an effective method to improve the curing and physicochemical properties of NR. By capping ZnO with BIAT and stearic acid, it becomes possible to save the extra time and energy required for these particles to diffuse onto the surface of ZnO via the viscoelastic rubber matrix. This provides a further improvement in acceleration of vulcanization and improvement in the physicomechanical properties of the resulting vulcanizates. The mixture containing optimum concentration of BIAT-capped-stearic acid-coated zinc oxide (ZOBS) has superior curing and physicomechanical properties compared with other homologues and the reference mixture containing uncapped ZnO. The increased crosslink density caused by the ZPS particles could increase the stiffness of vulcanizates containing ZPS. The capping technique could improve the scorch safety of rubber compounds by the delayed release of BIAT from the capped ZnO into the rubber matrix for interaction with CBS (conventional accelerator).

Sabura *et al.* [126] prepared nano zinc oxide by a solid-state pyrolytic method. Microscopic images and surface area studies showed that the prepared zinc oxide had particle sizes in the range 15–30 nm and surface area in the range 12–30 m^2^/g. The researchers used this nano zinc oxide as a curing agent in neoprene rubber. The optimum dosage of ZnO was found to be low compared with commercial ZnO. The cure characteristic and mechanical properties of the rubber were compared with those containing conventional ZnO. It was found that a low dosage of zinc oxide was enough to give equivalent curing and mechanical properties compared to neoprene rubber containing a higher dosage of commercial zinc oxide.

### 4.2. The Pharmaceutical and Cosmetic Industries

Due to its antibacterial, disinfecting and drying properties [127,128], zinc oxide is widely used in the production of various kinds of medicines. It was formerly used as an orally administered medicine for epilepsy, and later for diarrhoea. At the present time it is applied locally, usually in the form of ointments and creams, and more rarely in the form of dusting powders and liquid powders. ZnO has properties which accelerate wound healing, and so it is used in dermatological substances against inflammation and itching. In higher concentrations it has a peeling effect. It is also used in suppositories. In addition it is used in dentistry, chiefly as a component of dental pastes, and also for temporary fillings. ZnO is also used in various types of nutritional products and diet supplements, where it serves to provide essential dietary zinc [129].

For many years, before sun creams began to contain nanoparticles of ZnO or TiO_2_, they contained thick preparations which did not rub easily into the skin and which were cosmetically unattractive. Due to their ability to absorb UVA and UVB radiation, these products began to be used in creams. A new cream formula, containing a combination of ZnO and TiO_2_, solved the problem of an insufficiently white layer and produced a new medium which is more transparent, less adhesive and much more easily rubbed into the skin [130]. A number of studies have shown that titanium and zinc oxides are extremely good media in sun creams, since they absorb UV radiation, do not irritate the skin, and are easily absorbed into the skin [131–133].

### 4.3. The Textile Industry

The textile industry offers a vast potential for the commercialization of nanotechnological products. In particular, water repellent and self-cleaning textiles are very promising for military applications, where there is a lack of time for laundering in severe conditions. Also in the world of business, self-cleaning and water repellent textiles are very helpful for preventing unwelcome stains on clothes. Protection of the body from the harmful UV portion of sunlight is another important area. Many scientists have been working on self-cleaning, water repellent and UV-blocking textiles [134–140].

For textile applications, not only is zinc oxide biologically compatible, but also nanostructured ZnO coatings are more air-permeable and efficient as UV-blockers compared with their bulk counterparts [141]. Therefore, ZnO nanostructures have become very attractive as UV-protective textile coatings. Different methods have been reported for the production of UV-protecting textiles utilizing ZnO nanostructures. For instance, hydrothermally grown ZnO nanoparticles in SiO_2_-coated cotton fabric showed excellent UV-blocking properties [142]. Synthesis of ZnO nanoparticles elsewhere through a homogeneous phase reaction at high temperatures followed by their deposition on cotton and wool fabrics resulted in significant improvement in UV-absorbing activity [143]. Similarly, ZnO nanorod arrays that were grown onto a fibrous substrate by a low-temperature growth technique provided excellent UV protection [144].

Zinc oxide nanowires were grown on cotton fabric by Ates *et al.* [145] to impart self-cleaning, superhydrophobicity and ultraviolet (UV) blocking properties. The ZnO nanowires were grown by a microwave-assisted hydrothermal method and subsequently functionalized with stearic acid to obtain a water contact angle of 150°, demonstrating their superhydrophobic nature, which is found to be stable for up to four washings. The UV protection offered by the resulting cotton fabric was also examined, and a significant decrease in transmission of the UV range was observed. The self-cleaning activity of the ZnO nanowire-coated cotton fabric was also studied, and this showed considerable degradation of methylene blue under UV irradiation. These results suggest that ZnO nanowires could serve as ideal multifunctional coatings for textiles.

Research on the use of zinc oxide in polyester fibres has also been carried out at Poznan University of Technology and the Textile Institute in Lodz [146]. Zinc oxide was obtained by an emulsion method, with particles measuring approximately 350 nm and with a surface area of 8.6 m^2^/g. These results indicate the product’s favourable dispersive/morphological and adsorption properties. Analysis of the microstructure and properties of unmodified textile products and those modified with zinc oxide showed that the modified product could be classed as providing protection against UV radiation and bacteria.

### 4.4. The Electronics and Electrotechnology Industries

Zinc oxide is a new and important semiconductor which has a range of applications in electronics and electrotechnology [147–149]. Its wide energy band (3.37 eV) and high bond energy (60 meV) [150,151] at room temperature mean that zinc oxide can be used in photoelectronic [152] and electronic equipment [153], in devices emitting a surface acoustic wave [154], in field emitters [155], in sensors [156–161], in UV lasers [162], and in solar cells [163].

ZnO also exhibits the phenomenon of luminescence (chiefly photoluminescence—emission of light under exposure to electromagnetic radiation). Because of this property it is used in FED (field emission display) equipment, such as televisions. It is superior to the conventional materials, sulfur and phosphorus (compounds exhibiting phosphorescence), because it is more resistant to UV rays, and also has higher electrical conductivity. The photoluminescent properties of zinc oxide depend on the size of crystals of the compound, defects in the crystalline structure, and also on temperature [164–170]. ZnO is a semiconductor, and thin films made of that material display high conductivity and excellent permeability by visible rays. These properties mean that it can be used for the production of light-permeable electrodes in solar batteries. It also has potential uses as a transparent electrode in photovoltaic and electroluminescent equipment, and is a promising material for UV-emitting devices [171,172].

Zinc oxide is also used in gas sensors. It is a stable material whose weak selectivity with respect to particular gases can be improved by adding other elements. The working temperature of ZnO is relatively high (400–500 °C), but when nanometric particles are used this can be reduced to around 300 °C. The sensitivity of such devices depends on the porosity and grain size of the material; sensitivity increases as the size of zinc oxide particles decreases. It is most commonly used to detect CO and CO_2_ (in mines and in alarm equipment), but can also be used for the detection of other gases (H_2_, SF_6_, C_4_H_10_, C_2_H_5_OH). The zinc oxide used in the production of such equipment is obtained by a variety of methods (chemical vapour deposition, aerosol pyrolysis or oxidation of metallic zinc); it is important to control the process temperature, since this determines the properties of the product [173–175].

One of the most important applications of zinc oxide in electronics is in the production of varistors. These are resistors with a non-linear current-voltage characteristic, where current density increases rapidly when the electrical field reaches a particular defined value. They are used, among other things, as lightning protectors, to protect high-voltage lines, and in electrical equipment providing protection against atmospheric and network voltage surges. These applications require a material of high compactness, since only such a material can guarantee the stability and repeatability of the characteristics of elements made from it [176,177].

Certain unique electronic properties of ZnO are exploited in projection processes. The zinc oxide used for this purpose is produced from metallic zinc (from a suitable ore), so as to obtain a high-purity product. The photoconductor and semiconductor properties of ZnO are improved by thermal treatment, and also by the addition of other elements [178,179].

### 4.5. Photocatalysis

Intensive scientific work has taken place in recent years on photocatalysis. In this process, an electron-hole pair is produced below the intensity of light by means of oxidation or reduction reactions taking place on the surface of the catalyst. In the presence of a photocatalyst, an organic pollutant can be oxidized directly by means of a photogenerated hole or indirectly via a reaction with characteristic reactive groups (ROS), for example the hydroxyl radical OH·, produced in solution [180–182]. The most commonly used catalysts are TiO_2_ and ZnO. TiO_2_ exhibits photocatalytic activity below the intensity of UV light [183,184]. ZnO provides similar or superior activity to that of TiO_2_, but is less stable and less sensitive to photocorrosion [185]. Better stability, however, is provided by zinc oxide of nanometric dimensions, which offers better crystallinity and smaller defects [186]. The photocatalytic activity of ZnO can be further improved, and the range of the visible spectrum for zinc oxide can be extended, by adding other components [187].

The photocatalytic properties of zinc oxide, titanium dioxide and ZnO-TiO_2_ composite were investigated by Guo *et al.* [188]. ZnO was obtained in solution, this being a low-temperature and low-cost method. The properties and photocatalytic applications of the ZnO obtained in this way were studied. A sample was placed on a Petri dish containing an aqueous solution of methyl orange (pH 6.7). While being exposed to UV radiation the solution was mixed and stimulated by sunlight with or without polycarbonate filters. Absorption was measured immediately before exposure to UV and at set time intervals, using a UV/Vis spectrometer. These tests showed that the ZnO nanorods have similar photocatalytic properties (with UV) or slightly better properties (with stimulated sunlight) compared with TiO_2_ nanotubes. However, coating the surface of ZnO with a layer of TiO_2_ causes deterioration of the photocatalytic properties, possibly due to an increase in the quantity of defects. Summarizing their work, Guo *et al.* stated that the photocatalytic properties of ZnO can be influenced by coating with various substances and by the thickness of such coating.

Li *et al.* [189] also studied the photocatalytic properties of ZnO. ZnO nanospheres were obtained using an electrochemical method, in the presence of POMs (polyoxometalates), at room temperature. The experiments showed that POMs play a very important role in the formation of ZnO nanospheres. The photocatalytic properties of ZnO were determined using the example of degradation of rhodamine (RhB). Based on this study Li *et al.*, concluded that ZnO displays high photocatalytic activity below the UV range. The proposed simple, single-stage method of synthesis makes it possible to obtain spherical ZnO particles and provides the possibility of controlling their shape.

Ma *et al.* [190] demonstrated superior photocatalytic performance on ZnO nanorods and nanoflowers compared with commercial ZnO particles on methyl orange (MO). Besides organic dyes, UV-induced photocatalytic degradation of stearic acid by ZnO nanowires was also reported [191]. By the incorporation of dopants or formation of a composite with other materials, the photocatalytic properties of ZnO could be enhanced. Xu *et al.* [192] demonstrated improved photodegradation of MO by doping with cobalt on hydrothermally grown ZnO powders. One-dimensional heterostructures of ZnO and carbon nanofibres were reported to have significantly enhanced the photodegradation of rhodamine B compared with a pure ZnO counterpart [193]. It has also been reported that ZnO nanorod films can disinfect *E. coli* contaminated water with UV illumination [194].

Other studies by numerous researchers prove that ZnO offers unique photocatalytic properties, making it possible for this oxide to be used as a photocatalyst in the process of degradation of various substances [195–197].

### 4.6. Miscellaneous Applications

Apart from the applications mentioned above, zinc oxide can also be used in other branches of industry, including for example concrete production. The addition of zinc oxide improves the process time and the resistance of concrete to the action of water. Also, the addition of ZnO to Portland cement slows down hardening and quenching (it reduces the gradual evolution of heat), and also improves the whiteness and final strength of the cement.

Zinc oxide reacts with silicates (e.g., sodium silicate) to produce zinc silicates, which are water- and fire-resistant materials used as binders in paints. These fire-resistant and adhesive substances are used in the binding of cements used in the construction industry.

Methanol, the third most-important chemical product of chemical industry, is produced using a Cu/ZnO/Al_2_O_3_ catalyst, with small Cu particles promoted by their interaction with the ZnO substrate as the active component [198].

ZnO is also used for the production of typographical and offset inks. It imparts good printing properties (high fluidity). The addition of ZnO means that the inks have better covering power, pure shade and high durability, and prevents darkening. Zinc oxide is also used in pigments to produce shine.

It is added to many food products, including breakfast cereals. ZnO is used as a source of zinc, which is an essential nutrient. Thanks to their special chemical and antifungal properties, zinc oxide and its derivatives are also used in the process of producing and packing meat products (e.g., meat and fish) and vegetable products (e.g., sweetcorn and peas) [199].

As mentioned above, ZnO and its derivatives suppress the development and growth of fungi and moulds. Zinc oxide is added to fungicides to improve their effectiveness. Zinc oxide is also being used increasingly often as an animal feed additive, as it supports the correct growth of animals. It is also used as an artificial fertilizer [200].

Zinc oxide also has uses in criminology, in mechanical fingerprint analysis. It is also an ingredient in cigarette filters, as it selectively removes certain components from tobacco smoke. Filters are made of charcoal impregnated with ZnO and Fe_2_O_3_, which remove significant quantities of HCN and H_2_S from tobacco smoke without producing a smell. It also removes sulfur and its compounds from various liquids and gases, particularly industrial waste gases. Zinc also removes H_2_S from hydrocarbon gas, and desulfurizes H_2_S and other sulphur components.

ZnO and its derivatives are also used as an additive to car lubricating oils, reducing consumption and oxygen corrosion. Zinc oxide has also been used in various types of lubricants, such as those with EP additives, vibration-resistant lubricants and solid lubricants. In the future, advantage may also be taken of the adhesive properties of ZnO [201].

Because the compound is nontoxic, cheap, and chemically stable in the air, nanoparticles of zinc oxide can be used to make new eco-friendly substances for cell marking [202].

Recent advances in electrochemical biosensing based on a wide variety of nanostructures such as ZnO nanowires, nanotubes and nanoporous materials have attracted great interest in biosensor applications due to their remarkable properties such as non-toxicity, bio-safety, excellent biological compatibility, highelectron transfer rates, enhanced analytical performance, increased sensitivity, easy manufacture and low cost [203–205]. Moreover, ZnO has a highisoelectric point (*IEP*) of about 9.5, which can be expected to provide a positively charged substrate for immobilization of low-*IEP* proteins or enzymes such as uricase (*IEP* ~ 4.6) at a physiological pH of 7.4 [206,207]. In addition, ZnO has high ionic bonding (60%), and it dissolves very slowly at biological pH values [208].

Many researchers have attempted to correlate the biological activity of inorganic antibacterial agents with the size of the constituent particles [209,210]. Inorganic nanocrystalline metal oxides are particularly interesting because they can be prepared with extremely high surface areas, and are more suitable for biological molecular applications [211]. ZnO semiconductors have been extensively studied as antimicrobial agents due to their photocatalytic activity under UV light [212,213]. These antimicrobial substances based on inorganic chemicals have been found to be effective for therapy [214]. Padmavathy *et al.* [215] showed that ZnO nanoparticles were more abrasive than bulk ZnO (particle sizes in the range 0.1–1 μm), and this contributes to the greater mechanical damage to the cell membrane and the enhanced bactericidal effect produced by ZnO nanoparticles.

## 5. Conclusions

Zinc oxide is a multifunctional material because of its many interesting properties (piezo- and pyroelectric), a wide range of UV absorption, and high photostability, biocompatibility and biodegradability. ZnO can also be obtained with a variety of particle structures, which determine its use in new materials and potential applications in a wide range of fields of technology. Therefore the development of a method of synthesizing crystalline zinc oxide which can be used on an industrial scale has become a subject of growing interest in science as well as industry.

As can be seen from the survey of recent literature presented here, particles of zinc oxide—both nano- and micrometric—can be produced by many different methods. These can be divided into metallurgical and chemical methods. In metallurgical processes, zinc oxide is obtained by the roasting of a suitable zinc ore, via a direct or indirect process. Chemical methods can be divided into two groups: dispersion methods and condensation methods. In dispersion (mechanochemical) processes, zinc oxide is obtained by the grinding of suitable precursors. The resulting product may contain particles measuring approximately 20 nm. The condensation methods (controlled precipitation, the sol-gel method, hydro- and solvothermal methods, formation in an emulsion or microemulsion environment, and many others) involve the use of a molecularly homogeneous solution subjected to a process of nucleation.

The need to reduce the content of zinc oxide in certain materials, and to limit the degree of agglomeration, has led to the development of various methods of modifying the ZnO surface. Numerous reports in the literature indicate that modification processes can be carried out using inorganic substances (oxides and hydroxides), organic substances (alkoxysilanes, carboxylic acids), and certain polymer matrices, depending on how the systems obtained are to be used. Crystalline oxide powders, combined with other materials, provide possibilities for obtaining improved chemical, mechanical, optical or electrical properties.

Technology and knowledge relating to oxide materials of nano- and micrometric dimensions are currently among the most rapidly developing scientific and technological disciplines. The use of such materials can provide, among other things, more durable ceramics, transparent solar filters blocking infrared and ultraviolet radiation, and catalysts. These materials are also useful in biomedical research and in the diagnosis and treatment of diseases. They can be used to deliver medicines directly to diseased cells, in a way that avoids adverse effects.

The survey of the literature that has been given here shows that zinc oxide can be classed as a multifunctional material. This is thanks to such properties as high chemical stability, low electrical constant, high electrochemical coupling index, wide range of radiation absorption, and high photostability. It can be expected that interest in zinc oxide will continue to grow, and that this will lead to the development of new possibilities for its application.

## Figures and Tables

**Table 1.** Summary of methods of obtaining zinc oxide.

Method	Precursors	Synthesis conditions	Properties and applications	References
Mechanochemical process	ZnCl_2_, Na_2_CO_3_, NaCl	calcination: 2 h, 600 °C	hexagonal structure; particles diameter: 21–25 nm	[30]
		400–800 °C	hexagonal structure; particles diameter: 18–35 nm	[31]
		400 °C	regular shape of particles; diameter ~27 nm, *BET*: 47 m^2^/g	[32]
		0.5 h	particles diameter: 27–56 nm	[33]
		300–450 °C	particles diameter: ~51 nm, *BET*: 23 m^2^/g	[34]

Precipitation process	Zn(CH_3_COO)_2_, and KOH as a water solutions	temperature of process: 20–80 °C; drying: 120 °C	particles diameter: 160–500 nm, *BET*: 4–16 m^2^/g	[35]

	Zn(CH_3_COO)_2_, (NH_4_)_2_CO_3_, PEG10000 as a water solutions	drying: 12 h, 100 °C; calcination: 3 h, 450 °C	zincite structure; spherical particles (*D* ~ 30 nm); application: as a photocatlyst in photocatalytic degradation	[36]

	Zn(NO_3_)_2_	calcination: 2 h, 600 °C; aging: 240 h, 320 °C	wurtize structure; particles diameter: 50 nm; application: as a gas sensor	[37]

	Zn(NO_3_)_2_, NaOH	synthesis: 2 h; drying: 2 h, 100 °C	particles of spherical size of around 40 nm	[38]

	ZnSO_4_, NH_4_HCO_3_, ethanol	drying: overnight, 100 °C; calcination: 300–500 °C	wurtize structure; crystallite size 9–20 nm; particle size *D*: ~12 nm, *BET*: 30–74 m^2^/g	[39]

	Zn(CH_3_COO)_2_, NH_3_ aq.	precipitation temperature: 85 °C; drying: 10 h, 60 °C	hexagonal structure, shape of rods, flower-like particles: *L*: 150 nm, *D*: 200 nm	[40]

	ZnSO_4_, NH_4_OH, NH_4_HCO_3_	reaction: 30 min, 60 °C; drying: 12 h, 100 °C; calcination: 2 h, 400 °C	hexagonal structure, flake-like morphology (*D*: 0.1–1 μm, *L*: 60 nm)	[41]

	microsized ZnO powder, NH_4_HCO_3_	reaction: ~2 h, 25 °C; drying: 80 °C; calcination:1 h, 350 °C	hexagonal wurtize structure; flower-like and rod-like shape (*D*: 15–25 nm, *BET*: 50–70 m^2^/g)	[42]

	Zn(CH_3_COO)_2_, NaOH	reaction: 30 min, 75 °C; drying: overnight, room temperature	hexagonal structure; flower shape (*L*: >800 nm); application: antimicrobial activity	[43]

Precipitation in the presence of surfactants	ZnCl_2_, NH_4_OH, CTAB	aging: 96 h, ambient temperature, calcination: 2 h, 500 °C	zincite structure; particles diameter: 54–60 nm, *BET* = ~17 m^2^/g	[44]

	Zn(NO_3_)_2_, NaOH, SDS, TEA (triethanolamine)	precipitation: 50–55 min, 101 °C	wurtize structure, shape of rod-like (*L*: 3.6 μm, *D*: 400–500 nm) shape of nut-like and rice-like, size: 1.2–1.5 μm	[45]

Sol-gel	Zn(CH_3_COO)_2_, oxalic acid, ethanol and methanol	reaction temperature: 60 °C; drying: 24 h, 80 °C; calcination: 500 °C	zincite structure; aggregate particles: ~100 nm; shape of rod; particles *L*: ~500 nm, *D*: ~100 nm; *BET*: 53 m^2^/g; application: decontamination of sarin (neuro-toxic agent)	[46]

	Zn(CH_3_COO)_2_, oxalic acid (C_2_H_2_O_4_), ethanol	reaction: 50 °C, 60 min; dried of gel: 80 °C, 20 h; calcined: under flowing air for 4 h at 650 °C	hexagonal wurtize structure; uniform, spherically shaped of particles	[47]

	zinc 2-ethylhexanoate, TMAH ((CH_3_)_4_NOH), ethanol and 2-propanol	reaction: room temperature; drying: 60 °C	cylinder-shaped crystallites, *D*: 25–30 nm; *L*: 35–45 nm	[48]

	Zn(CH_3_COO)_2_, diethanolamine, ethanol	reaction: room temperature; annealed of sol: 2 h, 500 °C	hexagonal wurtize structure; particles: nanotubes of 70 nm	[49]

Solvothermal hydrothermal and microwave techniques	ZnCl_2_, NaOH	reaction: 5–10 h, 100–220 °C in teflon-lined autoclave	particles morphology: bullet-like (100–200 nm), rod-like (100–200 nm), sheet (50–200 nm), polyhedron (200–400 nm), crushed stone-like (50–200 nm)	[50]

	Zn(CH_3_COO)_2_, NaOH, HMTA (hexamethylenetetraamine)	reaction: 5–10 h, 100–200 °C; HMTA concentration: 0–200 ppm	spherical shape; particles diameter: 55–110 nm	[51]

	Zn(CH_3_COO)_2_, Zn(NO_3_)_2_, LiOH, KOH, NH_4_OH	reaction: 10–48 h, 120–250 °C	hexagonal (wurtize) structure, size of microcrystallites: 100 nm–20 μm	[52]

	Zn(CH_3_COO)_2_, NH_3_, zinc 2-ethylhexanoate, TMAH, ethanol, 2-propanol	time of autoclaving: 15 min, 2–72 h; final pH: 7–10	particles with irregular ends and holes; aggregates consist particles of 20–60 nm, *BET*: 0.49–6.02 m^2^/g	[53]

	trimethylamine N-oxide, 4-picoline N-oxide, HCl, toluene, ethylenediamine (EDA), N,N,N’,N’-tetramethylethylenediamine (TMEDA)	reaction: 24–100 h, 180 °C	wurtize structure; particles morphology: nanorods (40–185 nm), nanoparticles (24–60 nm)	[54]

Solvothermal hydrothermal and microwave techniques	Zn(CH_3_COO)_2_, Zn(NO_3_)_2_, ethanol, imidazolium tetrafluoroborate ionic liquid	reaction: 150–180 °C; drying: 80 °C in vacuum oven; calcinations: 500 °C	hexagonal (wurtize) structure, hollow microspheres (2–5 μm) consisted nano-sized particles and contained channels (10 nm); hollow microspheres consisted of nanorods (~20 nm); flower-like microspheres (2.5 μm)	[55]

	zinc acetylacetonate, methoxy-ethoxy- and n-butoxyethanol, zinc oximate	precursor concentration: 2.5–10 wt%; microwave heating: 800 W, 4 min; drying: 75 °C in air	zincite structure; average crystallite size: 9–31 nm; particles diameter: 40–200 nm; *BET*: 10–70 m^2^/g	[56]

	Zn(NO_3_)_2_, deionized water, HMT (hexamethylenetetramine)	microwave heating: 2 min, 90 °C; drying: 2 h, 60 °C	hexagonal wurtize structure, nanorod and nanowire shape (*L*: ~0.7 μm, *D*: ~280 nm); application: electronic and optoelectronic devices	[57]

Emulsion	Zn(NO_3_)_2_, surfactant (ABS, Tween-80 and 40, C_21_H_38_BrN)	reaction: 25 °C, pH~8; drying: 24 h, 80 °C; calcination: 2 h, 600 °C	grain size: cationic surfactants (40–50 nm), nonionic surfactants (20–50 nm), anonic surfactants (~20 nm)	[37]

	Zn(C_17_H_33_COO)_2_, NaOH, decane, water, ethanol	reaction: 2 h, room temperature or 90 °C	particles morphology: irregular particles aggregates (2–10 μm); needle-shaped (*L*: 200–600 nm, T: 90–150 nm); nearly spherical and hexagonal (*D*: 100–230 nm); spherical and pseudospherical aggregates (*D*: 150 nm)	[58]

	Zn(CH_3_COO)_2_, heptanes, Span-80, NH_4_OH	reaction: 1 h; aging: 2.5 h; drying: in rotary evaporator; calcination: 2 h, 700–1000 °C	hexagonal structure; spherical shape; particles diameter: 0.05–0.15 μm	[59]

	Zn(CH_3_COO)_2_, NaOH and KOH, cyclohexane, non-ionic surfactants	reaction: ambient temperature; drying: 24 h, 120 °C	hexagonal structure; particles morphology: solids (164–955 nm, *BET*: 8 m^2^/g), ellipsoids (459–2670 nm, *BET*: 10.6 m^2^/g), rods (396–825 nm, *BET*: 12 m^2^/g), flakes (220–712 nm, *BET*: 20 m^2^/g); crystallites size: 32–77 nm; application: as a photocatalyst	[60]

Microemulsion	Zn(NO_3_)_2_, NaOH, heptane, hexanol, Triton X-100, PEG400	reaction: 15 h, 140 °C; drying: 60 °C	hexagonal (wurtize) structure; particles morphology: needle (*L*: 150–200 nm, *D*: ~55 nm), nanocolumns (*L*: 80–100 nm, *D*: 50-80 nm), spherical (~45 nm)	[61]

Microemulsion	Zn(NO_3_)_2_, oxalic acid, isooctane, benzene, ethanol, diethyl ether, chloroform, acetone, methanol, Aerosol OT	reaction: 1 h; calcination: 3 h, 300 °C	equivalent spherical diameter: 11.7–12.9 nm, *BET*: 82–91 m^2^/g; grain size: 11–13 μm	[62]

	Zn(CH_3_COO)_2_, Aerosol OT, glycerol, C_20_H_37_NaO_7_S, n-heptane, NaOH, methanol, chloroform	reaction: 24 h, 60–70 °C; drying: 1 h, 100 °C; calcination: 3 h, 300–500 °C	hexagonal wurtize structure, spherical shape (15–24 nm), rods shape (*L*: 66–72 nm, *D*: 21–28 nm)	[63]

	ZnCl_2_, Zn(CH_3_COO)_2_, heptane, BTME (1,2-trimethoxysilyl)ethane, TMOS (tetramethoxysilane), methanol, Aerosol OT, NaOH	reaction: 2–3 h, room temperature or 40 °C; drying: under vacuum overnight; calcinations: 24 h, 700 °C	hexagonal structure, uniformly dispersed small particles, size of particles ~10 nm	[64]

Other method	Zn(CH_3_COO)_2_	thermal decomposition: 350–800 °C	uniform size of particles 20–30 nm	[65]

	Zn(NO_3_)_2_, deionized water, HMT (hexamethylenetetramine)	ultrasonic irradiation: 30 min, 80 °C; drying: 2 h, 60 °C	hexagonal wurtize structure, nanorod and nanowire shape (*L*: ~1 μm, *D*: ~160 nm); application: electronic and optoelectronic devices	[57]

	micron scale zinc metal powder	feed rate: 1 g/min; plasma power: 1 kW; O_2_ flow rate: 2.5 lpm; N_2_ flow rate: 12.5 lpm; reaction: 900 °C	nanowires shape (*L*: 1–30 μm, *D*: 5–50 nm) application: as hydrodesulfurization catalyst	[66]

	diethylzinc (DEZ), oxygen	helium as a carrier gas	wurtize structure; average particle size: 9 nm	[67]

Note: *BET*—surface area calculated based on *BET* equation; *D*—particles diameter; *L*—particles length.
